# Cyphers and cycles – A chemical basis of the differential attraction of mosquitoes to human odor

**DOI:** 10.1016/j.isci.2026.115575

**Published:** 2026-04-02

**Authors:** Annika Hinze, Anaïs Karine Tallon, Betelehem Wondwosen, Mengistu Dawit, Sharon Rose Hill, Björn Bohman, Rickard Ignell

**Affiliations:** 1 Unit of Chemical Ecology, Department of Plant Protection Biology, Swedish University of Agricultural Sciences, Alnarp, Sweden; 2 Department of Zoological Sciences, Addis Ababa University, Addis Ababa, Ethiopia

**Keywords:** Biological sciences, Entomology, Chemical Ecology, Chemical Biology, Chemistry

## Abstract

Anthropophilic mosquitoes, such as the yellow fever mosquito, *Aedes aegypti*, not only exhibit a robust host preference for humans but are also known to favor certain individuals over others, which has been attributed to variations in individual body odor. The underpinning chemical drivers, however, remain largely unidentified. Here, we assessed the differential attractiveness of 42 female participants to host-seeking *Ae. aegypti*, demonstrating that pregnancy or menstrual cycle phase significantly contributed to the individual level of attractiveness. Chemical and electrophysiological analyses of whole body odor samples identified 27 volatile organic compounds (VOCs) potentially involved in regulating the level of human attractiveness to mosquitoes. Behavioral assays further demonstrated that 1-octen-3-ol—present in the body odor of highly attractive participants and pregnant individuals—and the overall blend ratio of VOCs are sufficient to modulate mosquito preferences. Our findings suggest that multiple chemical motifs contribute to the heterogeneity in human attractiveness to mosquitoes.

## Highlights

• Individuals differ in their attractiveness to *Aedes aegypti* mosquitoes
• Mosquitoes detect a limited subset of human body odor compounds
• 1-Octen-3-ol levels are elevated in highly attractive and pregnant individuals
• Single odor compounds and odor blend ratio regulate differences in attraction

## Graphical abstract

**Figure:**
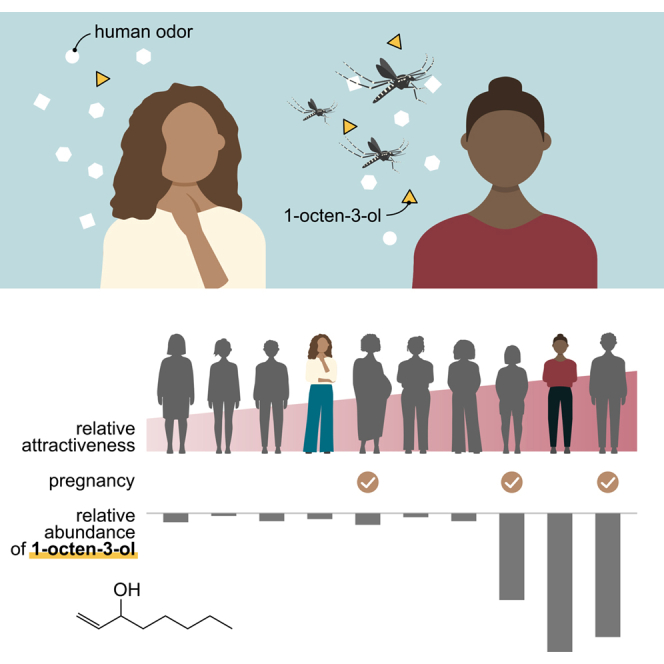

## Introduction

Some human individuals are more attractive to mosquitoes than others, a preference that is predominantly mediated by inter-individual differences in odor profiles.^1^^,^^2^^,^^3^^,^^4^^,^^5^^,^^6^^,^^7^^,^^8^ Mosquito preference for certain individuals has been attributed to a range of factors that are assumed to impact human body odor composition, such as genetics,^9^^,^^10^ ABO blood type,^11^^,^^12^^,^^13^^,^^14^ skin microbial composition,^15^ pregnancy,^16^^,^^17^^,^^18^ pathogen infection,^19^^,^^20^ sexual maturation,^21^ as well as personal care product use,^22^ diet,^23^ and alcohol consumption.^24^^,^^25^ Remarkably, even though differences in body odor have been recognized as the cause of individual variation in attractiveness to mosquitoes, and determining the contributing factors since the late 1950s, the underlying chemical mechanisms remain largely unknown. In particular, the identity and salient composition of key compounds in human body odor that drive this differential attractiveness to mosquitoes have yet to be fully elucidated.

The attraction of mosquitoes to individual human odor may differ due to variation in the abundance of attractive or aversive volatile organic compounds (VOCs) in human odor profiles.^7^^,^^9^^,^^26^^,^^27^^,^^28^ Differential attractiveness to mosquitoes is, however, likely not based upon the quantity and valence of single VOCs, but mediated by combinatorial coding, *i.e.*, the perception of VOC ratios within the human odor profile. Human odor is composed of hundreds, or even thousands, of different VOCs,^29^^,^^30^^,^^31^^,^^32^^,^^33^ associated with skin and breath, of which mosquitoes only detect a small proportion.^34^^,^^35^^,^^36^ Coding of human odor depends on the non-canonical co-expression of odorant receptors (ORs), involved in the detection of aldehydes, alcohols, aromatics, esters, ketones, and terpenes,^37^^,^^38^ as well as ionotropic receptors (IRs),^39^^,^^40^ responsible for the detection of carboxylic acids and amines.^41^^,^^42^ Knockout of neither the IR nor the OR pathway in mosquitoes abolishes their inter-individual human preference,^7^ emphasizing the complexity of the observed differential attractiveness. While a high abundance of select carboxylic acids has been associated with highly attractive individuals, other individuals do not fit this pattern.^7^ Similarly, other studies have identified select VOCs, e.g., octanal, decanal, 2-ethyl-1-hexanol, limonene, geranylacetone, and sulcatone, as possible determinants of individual attractiveness to mosquitoes, yet, the results are contradictory and many studies suffer from a low sample size.^9^^,^^26^^,^^28^^,^^43^^,^^44^ To date, while a few candidate VOCs have been suggested, it has not been possible to unequivocally identify key compounds or provide synthetic odor blends that reproduce the chemical signature of low and high attractiveness in individual persons.

The present study assessed the relative attractiveness of 42 female participants to the yellow fever mosquito, *Aedes aegypti*, and found that the menstrual cycle phase or pregnancy significantly contributed to the inter-individual variation in attractiveness. When stimulated with the headspace samples of individual participants, 27 VOCs were consistently detected by the antennae, or previously shown to be detected by the maxillary palps^45^ of host-seeking female mosquitoes. An analysis of similarity identified both the level of attractiveness and the menstrual cycle phase or pregnancy as explanatory variables for the variation in individual odor profiles. Indicator species analysis (ISA) further identified 1-octen-3-ol to be significantly more abundant in highly attractive, as well as in pregnant individuals. Synthetic blends mimicking the increased abundance of 1-octen-3-ol in the odor profiles of highly attractive individuals recapitulated the inter-individual host preference of the mosquitoes. Moreover, when tested in dual-choice assays, mosquitoes differentially discriminated among synthetic odor blends based on the average odor profile (ratio) of the least, average, and the most attractive individuals, suggesting that the chemical basis of individual attractiveness to mosquitoes is complex, and that several chemical motifs may contribute.

## Results

### Humans differ in relative attractiveness to *Ae. aegypti*

**Figure 1:**
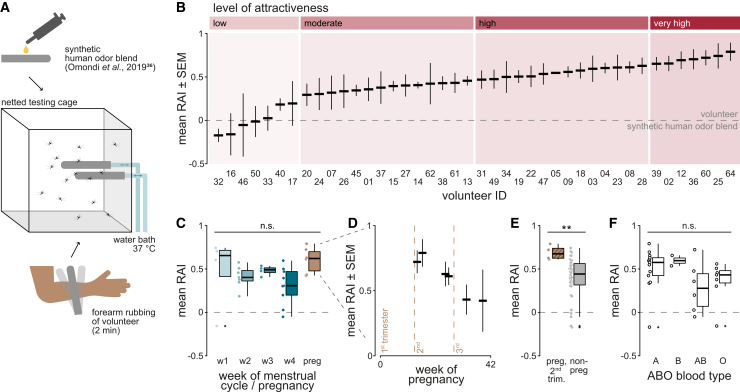
Relative attractiveness of human volunteers to female *Aedes aegypti* (A) Dual-choice landing assay. See also Table S1. (B) Mean relative attractiveness index (RAI) of individual volunteers to *Ae. aegypti* (15 mosquitoes per trial, three trials per volunteer) and their assigned level of attractiveness (top). Error bars denote standard error of the mean (SEM). (C) Mean RAI in relation to the week of menstrual cycle phase (w1-w4) or pregnancy (preg). Circles represent individual mean RAIs. Boxes represent upper and lower quartiles, whiskers denote 1.5 times interquartile distance, crosses outliers, and horizontal lines the median. Pairwise comparisons were made using a Dunn’s test with a Bonferroni correction. n(w1) = 4, n(w2) = 7, n(w3) = 4, n(w4) = 9, n(preg) = 6. (D) Mean RAI in relation to week of pregnancy. Error bars denote SEM. Three replicates per volunteer. (E) Mean RAI compares pregnant participants in their second trimester and non-pregnant individuals. n(pregnant, second trimester) = 4, n(non-pregnant) = 36. Asterisks indicate a statistically significant difference, tested with a Kruskal-Wallis rank-sum test. p = 0.0060. (F) Mean RAI in relation to the ABO blood type. n(A) = 13, n(B) = 2, n(AB) = 6, n(O) = 7.

To test differences in the attractiveness of individual participants, their odor was collected on glass rods and tested against a synthetic human odor control using a dual-choice landing assay (Figure 1A). The 42 participants were divided into four groups (*i.e*., low, moderate, high, and very high) according to their mean relative attractiveness index (RAI; Figure 1B) by quartile normalization. The menstrual cycle phase or pregnancy contributed significantly (Kruskal-Wallis test, *p* = 0.049) to the observed RAI. However, pairwise comparisons between the menstrual cycle weeks or pregnancy did not reveal any significant differences (Dunn’s test, *p* > 0.06; Figure 1C). Nonetheless, pregnant participants in their second trimester of pregnancy were more attractive than participants in their third trimester (*p* = 0.064; Figure 1D), and significantly more attractive than non-pregnant individuals (*p* = 0.0060; Figure 1E). Neither the ABO blood type (*p* = 0.16; Figure 1F), the use of hormonal contraception (*p* = 0.90), nor the age of the participant (*p* = 0.44; range 20–37 years) was found to affect the relative attractiveness to mosquitoes.

### Mosquitoes detect a narrow range of human body odors

**Figure 2:**
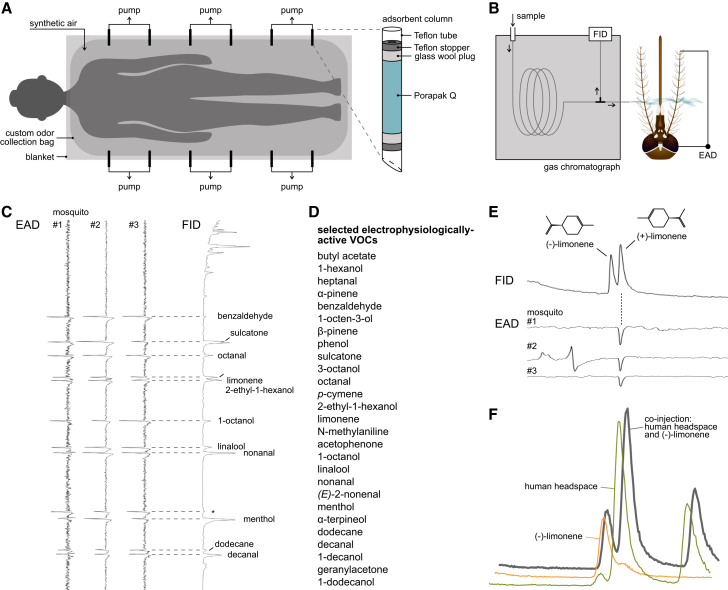
Combined gas chromatography and electroantennographic detection (GC-EAD) analysis of human headspace samples (A) Schematic of the headspace odor collection setup. (B) Schematic of the GC-EAD setup. FID: Flame ionization detector. (C) Representative GC-EAD responses of antennal preparations of female *Aedes aegypti* to the headspace extract of a human volunteer. Three biological replicates are shown. The asterisk indicates a compound that elicited an electrophysiological response in fewer than three human headspace samples, and therefore did not meet the inclusion criteria. See also Figure S1. (D) List of electrophysiologically active volatile organic compounds (VOCs) present in human headspace extracts selected for quantification. The VOCs are ordered by retention time. (E) Representative GC-EAD responses of *Ae. aegypti* to a racemic mixture of limonene, injected on a chiral column, demonstrating enantiomeric selectivity of the antenna. (F) Extracted ion chromatograms (m/z = 93) of a pooled human headspace extract (blue), an injection of (−)-limonene (yellow), and the co-injection of both (dark gray), run on a combined gas chromatography and mass spectrometry instrument equipped with a chiral column. First peak: (−)-limonene, second peak: (+)-limonene, third peak: sulcatone. The co-injected (−)-limonene does not enhance the dominant limonene peak of the human headspace extract. See also Table S3.

Following the collection of human headspace samples (Figure 2A), combined gas chromatography and electroantennographic detection (GC-EAD) and GC and mass spectrometry (GC-MS) analyses identified 26 VOCs that elicited a physiological response in the antennal preparations of female *Ae. aegypti* (Figures 2B–2D; Figure S1). In addition, 1-octen-3-ol, which is detected by the maxillary palps,^45^ was identified in the human skin emanations and added to the panel, bringing the total number of investigated VOCs to 27. The antenna of the female *Ae. aegypti* displayed enantiomeric selectivity for chiral compounds found in human headspace extracts: (+)-α-pinene, (−)-β-pinene, (−)-2-ethyl-1-hexanol, (+)-limonene, (+)-linalool, and (−)-menthol (Figure 2E; Table S3). The human headspace extracts mostly contained both enantiomers of these compounds, although usually not as racemates (Figure 2F; Table S3). Quantification of the panel of 27 electrophysiologically active VOCs, using GC-MS analysis, revealed no correlation between the total amount of extracted VOCs and the relative attractiveness level of the individual participants (Kruskal-Wallis, *p* = 0.32; Figure S2). Further analyses focused, therefore, on the relative abundance of VOCs.

### 1-Octen-3-ol is elevated in highly attractive and pregnant individuals

**Figure 3:**
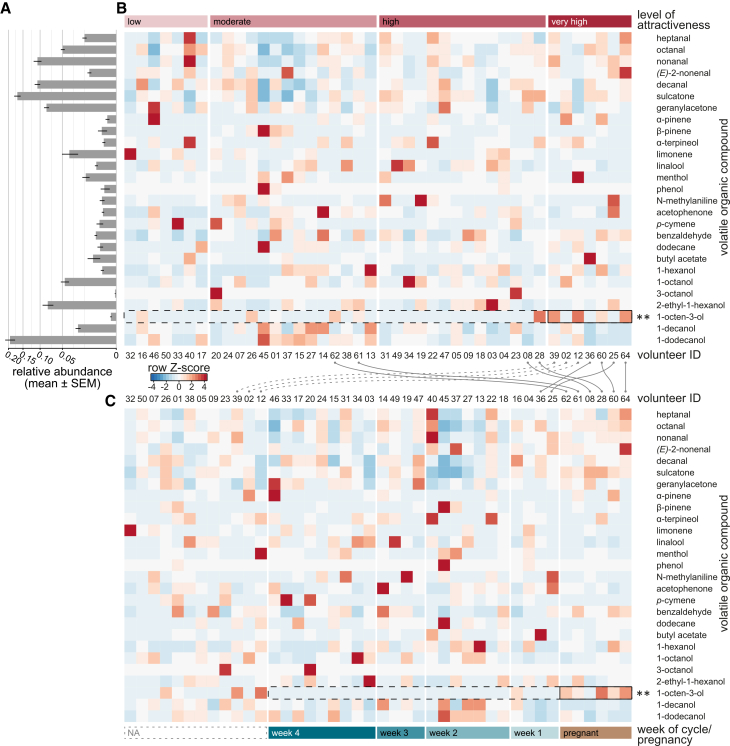
Human odor profiles in relation to level of attractiveness and week of the menstrual cycle or pregnancy (A) Mean relative abundance of the selected panel of volatile organic compounds (VOCs). Bars denote standard error of the mean (SEM). (B and C) Heat plots display the divergence of VOC abundance from the mean for each volunteer in relation to (B) the level of attractiveness and (C) the week of the menstrual cycle or pregnancy. Blue color indicates a lower abundance and red a higher abundance than the mean (row *Z* score). Gray lines highlight the change in ranking for very highly attractive and pregnant participants (see also Figure S4). NA refers to participants who did not (or could not) share information about the week of their menstrual cycle or pregnancy. Asterisks and black boxes denote compounds with significantly higher similarity of VOC abundance within (ISA), rather than between groups (ANOSIM). p(attractiveness; 1-octen-3-ol) = 0.0063, p(cycle/pregnancy; 1-octen-3-ol) = 0.001. See also Figure S3.

The six most abundant VOCs associated with human skin emanations were, in order of abundance, 1-dodecanol, sulcatone, decanal, nonanal, geranylacetone, and 2-ethyl-1-hexanol (Figure 3A), although their individual relative abundance was not correlated with the level of attractiveness (ISA, *p* > 0.05; see also Figure S3). The level of attractiveness (ANOSIM, R = 0.05, *p* = 0.030) and the menstrual cycle phase or pregnancy (R = 0.15, *p* = 0.021) explained the variation in odor profiles, indicating that individuals with different attractiveness levels have slightly different odor profiles overall. In contrast, we did not find any clear, consistent differences in full VOC profiles between groupings of ABO blood type (R = −0.08, *p* = 0.81) and use of hormonal contraception (R = −0.14, *p* = 0.94). Our analysis (*e.g*., negative ANOSIM R values) indicates that samples within the same “group” (*i.e*., level) are more dissimilar from each other than they are from samples in other groups, suggesting that these group labels may not reflect any meaningful structure in the odor profiles. However, certain key compounds varied in meaningful and biologically relevant ways, as shown per ISA. High 1-octen-3-ol levels were more common in the odor of the most attractive, as well as pregnant, individuals (Figures 3B and 3C), and significantly associated with both high attractiveness (ISA; *p* = 0.0063) and pregnancy (*p* = 0.001). While there is overlap between the two groups, most highly attractive participants were not pregnant (Figure S4). No VOCs were associated with any other level of attractiveness or the week of the menstrual cycle. The use of hormonal contraception was significantly associated with N-methylaniline (*p* = 0.0062) and dodecane (*p* = 0.013), whereas not using hormonal contraception was significantly associated with 1-octen-3-ol (*p* = 0.040). No VOCs were significantly associated with any specific ABO blood type.

### Single VOCs and blend ratios regulate inter-individual differences in attraction

**Figure 4:**
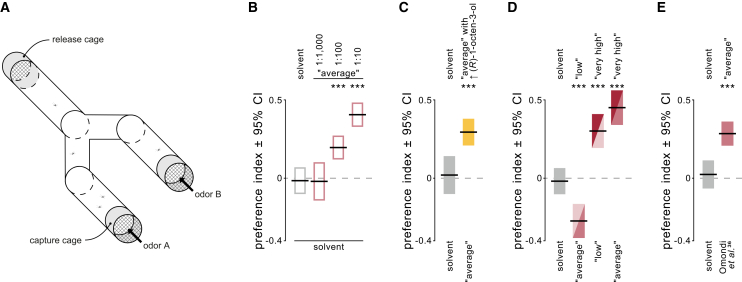
Behavioral response of female *Aedes aegypti* to a panel of synthetic human blends (A) Dual-choice olfactometer. (B) Mean preference index for an increasing concentration of the “average” blend against a solvent control. Five mosquitoes per trial; *n* = 21–31. (C) Mean preference index of the modification of the “average” blend to include a higher relative abundance of (*R*)-1-octen-3-ol, as found in highly attractive individuals. *n* = 10–29. (D and E) Mean preference index of (D) blends based on the different levels of attractiveness, and (E) the “average” blend tested against the synthetic human odor blend from Omondi et al.^36^ Colors according to the level of attractiveness (see *e.g*., Figure 3B). *n* = 21–42. Black horizontal lines are the mean with 95% confidence interval (vertical bars). Asterisks indicate a statistical difference from zero, *i.e.*, a preference, tested with Bonferroni-corrected one-sample two-tailed Wilcoxon tests. See also Tables S1, S5, and S6.

Based on the panel of 27 VOCs detected and identified by GC-EAD and GC-MS analyses of the human skin emanations, several synthetic blends were designed and tested in a dual-choice olfactometer (Figure 4A). Mosquitoes exhibited a dose-dependent attraction to the “average” blend, and a significant preference for concentrations of the “average” blend stock solution higher than 1:100 (Wilcoxon test, *p* < 0.00065; Table S1) when compared to a blank (Figure 4C). The dose of 1:100 (*p* = 0.00065) was selected for consecutive experiments. Adapting the relative abundance of (*R*)-1-octen-3-ol of the “average” blend to match the higher abundance of this compound, as found in highly attractive individuals (see Figure 3A), significantly affected the attractiveness of the blend (Kruskal-Wallis rank-sum test, *p* = 0.0025; Figure 4C), with mosquitoes significantly preferring the blend with an increased relative abundance of (*R*)-1-octen-3-ol (Wilcoxon test; *p* = 0.00012; from 0.05% to 0.3%). Synthetic blends mimicking the VOC profiles of the different attractiveness levels recapitulated the observed ranking: While the blend based on low attractiveness was significantly less attractive than the “average” blend (Wilcoxon test; *p* = 0.00052), the blend based on very high attractiveness was preferred to both the “low” (*p* = 0.00034) and “average” (*p* < 0.0001) blends (Figure 4D). Furthermore, the “average” blend was significantly more attractive than the synthetic human odor blend from Omondi et al.^36^ (*p* = 0.00015; Figure 4E), which was used to test the relative attractiveness of the participants (see Figures 1A and 1B).

## Discussion

Host-seeking mosquitoes of several species show a robust, odor-mediated preference for certain human individuals over others when several humans are present in their environment.^1^^,^^2^^,^^3^^,^^4^^,^^5^^,^^6^^,^^7^^,^^8^ The factors contributing to the observed heterogeneity in attractiveness have been studied since the 1950s,^9^^,^^10^^,^^11^^,^^12^^,^^13^^,^^14^^,^^15^^,^^16^^,^^17^^,^^18^^,^^19^^,^^20^^,^^21^^,^^22^^,^^23^^,^^24^^,^^25^ but the chemical drivers remain largely unclear. This study demonstrated inter-individual differences in the relative attractiveness of female volunteers to *Ae. aegypti*, and identified pregnancy or menstrual cycle phase as a determining factor. Analyses of the headspace samples of the volunteers and the synthetic human odor blend used for the attractiveness assay identified a narrow range of VOCs to be consistently detected by the peripheral olfactory system of *Ae. aegypti*. Both the level of attractiveness and the menstrual cycle phase or pregnancy were explanatory for the variation in odor profiles. Behavioral experiments demonstrated that, while inter-individual discrimination may rely on the detection of select VOCs, *i.e*., (*R*)-1-octen-3-ol, the context, including qualitative and quantitative differences in blend composition, is of equal importance in regulating the level of attraction.

### (*R*)-1-octen-3-ol as a signature volatile of high attractiveness

By comparing the odor profiles of highly and poorly attractive individuals, and testing derived synthetic blends, we identified 1-octen-3-ol, which was a compound of low abundance in the human odor profile, as a signature VOC of high attractiveness to mosquitoes. The function of this VOC in mosquitoes is well-characterized, as it is present in vertebrate odor,^36^^,^^45^^,^^46^^,^^47^^,^^48^ likely involved in inter-specific host discrimination,^45^^,^^49^^,^^50^ and a common attractant in commercial mosquito traps.^51^ Mosquitoes show molecular, physiological, and behavioral enantiomeric specificity to (*R*)-1-octen-3-ol,^49^^,^^52^^,^^53^^,^^54^^,^^55^ which is predominantly derived from the enzymatic oxidative breakdown of linoleic acid^56^ and widely found in the odor of fungi,^57^^,^^58^ as well as produced by a few groups of cyanobacteria and plants.^59^^,^^60^ On human skin, linoleic acid is present in the sebaceous glands, where it is both oxidized to squalene, an important component of human sebum, and secreted as a skin surface lipid.^61^^,^^62^^,^^63^ The microbiome of sebaceous glands and of the skin surface may play an important role in degrading linoleic acid to (*R*)-1-octen-3-ol, however, the biosynthetic pathway remains unclear. Thus, while inter-individual differences in (*R*)-1-octen-3-ol abundance are likely due to diverging skin microbiomes,^64^ genetic or physiological differences in sebum production may play an additional role. Although individuals with a high relative abundance of 1-octen-3-ol often ranked among the most attractive participants, this VOC was not consistently enriched across all highly attractive individuals. This suggests that 1-octen-3-ol may not be the only compound to drive high attractiveness, and its effect may depend on the presence or abundance of other compounds in the odor profile. Instead, our findings point to complex and multi-layered olfactory processing in which multiple chemical classes—and their specific ratios and combinations—contribute to individual attractiveness. This may include certain carboxylic acids, which have previously been linked to high individual attractiveness in many, but not all, highly attractive persons.^7^ Carboxylic acids were not included in this study due to their unfavorable GC properties. (*R*)-1-octen-3-ol is detected by the OR pathway.^50^^,^^53^^,^^54^ Interestingly, mosquitoes lacking the expression of the OR co-receptor *Orco*, and thereby the functionality of the OR pathway, retain attraction to humans, but lose the ability to discriminate between human and non-human hosts.^50^ However, *Orco* mutants are able to detect differences between humans of different attractiveness levels.^7^ Likewise, the knockout of IR co-receptors may diminish general attraction, but does not impair individual human preference.^7^ The fact that the knockout of neither the OR nor the IR pathway in mosquitoes abolishes their ability to discriminate between humans^7^ further supports the concept that mosquito preference is not based on a single compound or pathway. This redundancy may be mirrored at the cellular level, as OR8, which detects (*R*)-1-octen-3-ol,^54^ shows co-expression with several co-receptors of the IR pathway.^39^ It is also possible to hypothesize that the addition of certain background VOCs could diminish or mask the effect of otherwise attractive compounds. Overall, high attractiveness to mosquitoes emerges from a multifaceted interplay of volatiles, rather than from the presence of any single “attractive” molecule.

### Pregnancy as a driver of host preference

Previous work demonstrated that pregnant individuals attract up to twice as many host-seeking malaria vectors (*i.e*., number of individuals) as non-pregnant individuals, thereby increasing their risk of infection substantially.^16^^,^^17^^,^^18^ This difference in attractiveness is hypothesized to be attributed to the higher breathing frequency, and thus higher amounts of emitted carbon dioxide and breath-related compounds, as well as a higher body temperature observed during pregnancy.^16^^,^^17^ In addition, we demonstrated that the yellow fever vector *Ae. aegypti* is strongly attracted to most pregnant individuals, and the VOCs associated with their body odor, *i.e*., 1-octen-3-ol, play an important role in regulating attraction. The selective attraction to certain pregnant individuals appears to be linked to the gestational stage: While pregnant participants in their second trimester were highly attractive to *Ae. aegypti*, participants in late pregnancy were of average attractiveness. There is, however, no clear correlation with the relative abundance of 1-octen-3-ol, suggesting a redundancy in signaling, in which other VOCs may play a contributing role. As previous studies^16^^,^^17^^,^^18^ did not record or analyze the gestational age, future research will show if high attractiveness in mid-pregnancy and average attractiveness in late pregnancy may be a general feature or an effect of low sample size. While sebum secretion is generally increased during pregnancy as a result of higher levels of the neurohormone prolactin,^65^ it is unclear if sebum composition is modulated according to the gestational age. Furthermore, despite the human nose detecting the modulation of body odor by the menstrual cycle,^66^^,^^67^^,^^68^ mosquitoes did not show a clear preference for the odor of participants in any week of the menstrual cycle, regardless of hormonal contraceptive use, implying that mosquito preference may rely on a different subset of VOCs than those modulated by the cycle. Sampling the body odor of the same individuals throughout their cycle would be valuable to test this hypothesis.

### Blend context and ratio-dependent attraction

Next to preferring a blend with an increased relative abundance of one single compound, host-seeking mosquitoes also discriminated between blends mimicking the odor profile of different attractiveness levels, thereby recapitulating the assigned ranking. Recent studies in mosquitoes have demonstrated the importance of the overall blend composition and ratio in resource selection and discrimination,^36^^,^^69^^,^^70^^,^^71^^,^^72^ aligning with previous findings in other insects.^73^ Effective discrimination among blends of ubiquitous compounds requires a sensory system that accommodates variations in host odor profiles while maintaining the specificity needed to distinguish hosts from non-hosts.^73^ This specificity may be achieved by the synergism of VOCs present in the blends of suitable resources that are detected simultaneously and thus encoded by their close temporal association.^71^^,^^74^^,^^75^ Blends that are skewed in their ratios or omit certain compounds often lose their attractiveness to mosquitoes when tested in ecologically relevant concentrations.^70^^,^^72^ At the same time, flexibility in host recognition may be accomplished by a certain level of redundancy,^73^ at which multiple chemical motifs signal host identity.^36^

Taken together, (*R*)-1-octen-3-ol, a breakdown product of human sebum, appears to be closely associated with individual attractiveness, suggesting that it may provide a reliable cue to indicate a suitable host for mosquitoes. This is supported by other studies highlighting the role of human sebum-derived VOCs in inter-individual differences in human attractiveness, such as select carboxylic acids.^7^ Additional studies have shown the correlation between high attractiveness and the presence of sebum-derived VOCs, such as octanal, decanal, sulcatone, and geranylacetone, found in our study, as well as lactic acid and other carboxylic acids.^8^^,^^9^^,^^28^^,^^76^ Moreover, human sebum-derived VOCs may also play a key role in the discrimination of human and non-human hosts.^27^^,^^44^^,^^45^^,^^48^^,^^63^^,^^77^^,^^78^ To build on these findings, future studies and behavioral assays (*e.g*., odor blends containing (*R*)-1-octen-3-ol in combination with other sebum-derived VOCs) could aim to examine synergistic or antagonistic effects among compounds.

### Closing remarks

Mosquito preference for individual humans is odor-mediated and non-random, with strong implications for the individual risk of infection with mosquito-borne diseases. Intensive research has focused on identifying the chemical basis of differential attractiveness, determining not only candidate compounds and motifs, but also demonstrating the complexity of how these VOCs are encoded by the mosquito olfactory system. Future studies are required to identify both the proximate and ultimate mechanisms regulating how and why mosquitoes differentiate between humans, *i.e.*, to address the question if inter-individual preference is a trait favored by selection or a by-product of an olfactory discrimination task linked to another context. Our results provide a foundation for future behavioral and physiological studies aimed at understanding the odor coding underlying differential attractiveness to mosquitoes. Moreover, the identification of a disease vector-targeted synthetic human odor may provide a basis for future control strategies.

### Limitations of the study

The current study suggests that (*R*)-1-octen-3-ol may be crucial to *Ae. aegypti* in regulating their preference for certain individuals over others. Although this VOC is a known attractant for many other mosquito species,^33^ it remains unclear whether variation in its relative abundance across individuals translates into the observed heterogeneity in human attractiveness to other mosquito species.^1^^,^^2^^,^^3^^,^^4^^,^^5^^,^^8^^,^^9^ Moreover, as our study used a long-established laboratory strain, the valence of (*R*)-1-octen-3-ol for *Ae. aegypti* would need to be confirmed with natural populations. Other limitations include the modest sample size, especially of pregnant individuals, which may amplify unknown confounding factors, such as a potential bias in the selection of volunteers. Our study was further limited by the headspace collection method used: Human odor is a complex blend of VOCs spanning multiple chemical classes, and no single odor collection or analysis method can account for all of them. Overall, the present study is an important step toward understanding the chemical mechanisms that drive the evident heterogeneity in the attractiveness of individuals to mosquitoes, as well as takes previously neglected factors, such as pregnancy and menstrual cycle phase, into account.

## Resource availability

### Lead contact

Requests for further information and resources should be directed to and will be fulfilled by the lead contact, Rickard Ignell (rickard.ignell@slu.se).

### Materials availability

This study did not generate new unique reagents.

### Data and code availability

• De-identified data supporting these analyses have been deposited at the Swedish National Data Service repository at https://doi.org/10.5878/vh3n-xm78 and are publicly available as of the date of publication. Accession numbers are listed in the key resources table.
• This paper does not report original code.
• Any additional information required to reanalyze the data reported in this paper is available from the lead contact upon request.

## Acknowledgments

We would like to thank Göran Birgersson for his support in setting up the odor collection and advice on GC-MS, as well as the participants for their interest and time. This project was supported by funding by 10.13039/501100004359Vetenskapsrådet.

## Author contributions

Conceptualization, A.H., A.K.T., S.R.H., and R.I.; data curation, A.H., A.K.T., and B.W.; formal analysis, A.H., A.K.T., and B.W.; funding acquisition, R.I.; investigation, A.H., A.K.T., B.W., and M.D; methodology, A.H., A.K.T., B.B., and R.I.; project administration, A.H., A.K.T., and R.I.; visualization, A.H.; writing – original draft, A.H., S.R.H., and R.I.; writing – review and editing, all authors.

## Declaration of interests

The authors declare no competing interests.

## STAR★Methods

### Key resources table

**Table:** 

REAGENT or RESOURCE	SOURCE	IDENTIFIER
**Chemicals, peptides, and recombinant proteins**		

1-Decanol (decan-1-ol)	Fluka	30600(A52605)
1-Dodecanol (dodecan-1-ol)	Fluka	417504
1-Hexanol (hexan-1-ol)	Sigma-Aldrich	102296086
1-Octanol (octan-1-ol)	Fluka	293245
(*R*)-1-Octen-3-ol ((3*R*)-oct-1-en-3-ol)	Sigma-Aldrich	102402758
2-Ethyl-1-hexanol (2-ethylhexan-1-ol)	Fluka	04050/2032343
2-Nonanol (nonan-2-ol)	Sigma-Aldrich	102400254
(*E*)-2-Nonenal ((*E*)-non-2-enal)	Sigma-Aldrich	07097MJ-468
3-Octanol (octan-3-ol)	Fluka	1003218664
Acetophenone (1-phenylethanone)	Acros Organics	102412500
(+)-α-pinene ((1*R*,5*R*)-4,6,6-trimethylbicyclo[3.1.1]hept-3-ene)	Sigma-Aldrich	08926AE
(−)-α-pinene ((1*S*,5*S*)-2,6,6-trimethylbicyclo[3.1.1]hept-2-ene	Sigma-Aldrich	1002438062
(+)/(−)-α-pinene (2,6,6-trimethylbicyclo[3.1.1]hept-2-ene)	Sigma-Aldrich	1001248567
(−)-α-terpineol (2-[(1*S*)-4-methylcyclohex-3-en-1-yl]propan-2-ol)	Merck	1003076249
Benzaldehyde	Sigma-Aldrich	102436873
(+)-β-pinene ((1*R*,5*R*)-6,6-dimethyl-2-methylidenebicyclo[3.1.1]heptane)	Supelco	102365315
(−)-β-pinene ((1*S*,5*S*)-6,6-dimethyl-2-methylidenebicyclo[3.1.1]heptane)	Sigma-Aldrich	00307LG-437
Butyl acetate	Sigma-Aldrich	1003263101
Decanal	Sigma-Aldrich	102018838
Dodecane	Fluka	K2324-162 14-14
Geranylacetone ((5*E*)-6,10-dimethylundeca-5,9-dien-2-one)	Alfa Aesar, SAFC	07937A-A19184, 082968H
Helium	Strandmöllen	185055S
Heptanal	Sigma-Aldrich, SAFC	534525-506, 102409571
Hexane	Merck	K50829591-845
(+)-Isomenthol ((1*S*,2*R*,5*R*)-5-methyl-2-propan-2-ylcyclohexan-1-ol)	PhytoLab	PHL80016
(+)-Limonene ((4*R*)-1-methyl-4-prop-1-en-2-ylcyclohexene)	Sigma-Aldrich	102413721
(−)-Limonene ((4*S*)-1-methyl-4-prop-1-en-2-ylcyclohexene)	Sigma-Aldrich	1002027132
(+)/(−)-Linalool (3,7-dimethylocta-1,6-dien-3-ol)	Sigma-Aldrich	102377664
(−)-Linalool ((3*R*)-3,7-dimethylocta-1,6-dien-3-ol)	Fluka	GA100035
(+)-Menthol ((1*S*,2*R*,5*S*)-5-methyl-2-propan-2-ylcyclohexan-1-ol)	Sigma-Aldrich	224464
(−)-Menthol ((1*R*,2*S*,5*R*)-5-methyl-2-propan-2-ylcyclohexan-1-ol)	Acros Organics	12540
*m*-cymene (1-methyl-3-propan-2-ylbenzene)	Supelco	255289/30037
Nerylacetone ((5*Z*)-6,10-dimethylundeca-5,9-dien-2-one)	Fluka	72185
N-methylaniline	AK Scientific	TC45824C/30023
Nonanal	Fluka	1003315907
Octanal	Fluka	1003266587
*o*-cymene (1-methyl-2-propan-2-ylbenzene)	Sigma-Aldrich	255270
*p*-cymene (1-methyl-4-propan-2-ylbenzene)	Sigma-Aldrich	102070990
Pentane	Supelco	1.00882.1000
Phenol	Sigma-Aldrich	101985219
Synthetic air (20.9% O_2_, 79.1% N_2_)	Strandmöllen AB	0027613778000010
Sulcatone (6-methylhept-5-en-2-one)	Sigma-Aldrich	1003347793

**Deposited data**		

(1) human data, including relative amounts of target compounds, and (2) two-choice assay data	This paper	https://doi.org/10.5878/vh3n-xm78

**Experimental models: Organisms/strains**		

*Aedes aegypti* (Rockefeller strain)	Established colony at SLU since 2004 from eggs kindly provided by Prof. Ring Cardé, UC Riverside	NA

**Software and algorithms**		

AMDIS 32	National Institute of Standards and Technology	https://chemdata.nist.gov/dokuwiki/doku.php?id=chemdata:amdis
R^79^	The R Foundation	https://www.r-project.org/
GcEAD/2015 (GC-EAD software), version 1.2.3	Syntech	https://github.com/ellis/gcead?tab=readme-ov-file

**Other**		

BugDorm cages (30 cm × 30 cm × 30 cm; 17.5 cm × 17.5 cm × 17.5 cm)	MegaView Science	DP1000; BD4S1515
Flow meter	Kytala Instruments	NA
Luxmeter	LT-Lutron	E39583
Membrane feeder	Hemotek Ltd	NA
Non-perfumed soap (“Special care mild tvål”)	Apotek Hjärtat	7340160907522
Porapak Q (80–100 mesh)	Alltech Associates	128312
Pumps	KNF	NMP830KNDC
Roasting tubes (180 cm × 60 cm)	Melitta/Toppits	3322281106723
Teflon tubing (3 mm inner diameter)	Habia Teknofluor	30.05477
TetraMin fish food	Tetra GmbH	DE-N!400080

### Experimental model and study participant details

#### Mosquito rearing

*Aedes aegypti* (Rockefeller strain) were reared at 27 ± 1°C and 65 ± 5% relative humidity under a 12 h light: 12 h dark regimen. Mosquito larvae were fed daily on TetraMin fish food (Tetra GmbH, Melle, Germany). Pupae were collected and transferred to BugForm cages (30 cm × 30 cm × 30 cm or 17.5 cm × 17.5 cm × 17.5 cm; MegaView Science, Taichung City, Taiwan) to emerge, and adult mosquitoes provided with *ad libitum* access to 10% sucrose. For colony maintenance, female mosquitoes were offered sheep blood (Håtunalab AB, Bro, Sweden) using a membrane feeder (Hemotek Ltd, Blackburn, UK). For both the behavioral assays and combined gas chromatography and electroantennographic detection (GC-EAD) analysis, non-blood-fed females, which were co-housed with males since eclosion, were tested at 5–6 days post-emergence (dpe).

#### Human participants

Participants in this study (*n* = 42) identified as female, were 20–37 years old (mean 29.2 years) and came from multiple ethnic backgrounds and nationalities. None of them used tobacco products. All participants provided written informed consent prior to participation (see Data S1). Participants were asked for information on their ABO blood type, menstrual cycle or pregnancy, and use of hormonal contraception, of whom 67%, 71%, and 92%, respectively, agreed to share. Starting 24 h before testing, the relative attractiveness of the individual participants to mosquitoes and collection of their body odor, they were asked to strictly follow rules regarding diet, use of cosmetics, and lifestyle modified from.^66^ Participants were instructed to (1) not use any perfumes, deodorants, conventional shower products, or other cosmetics that contained perfumes, as well as any products that could interfere with body odor production, such as antiperspirants. A non-perfumed soap (special care mild tvål, Apotek Hjärtat, Solna, Sweden) was provided. Moreover, participants were asked to (2) refrain from eating garlic, onion, spicy food, blue cheese, vinegar, cabbage, radish, fermented milk products, or marinated fish. In addition, participants should not (3) drink any alcohol or consume any other drugs, (4) engage in any intense physical activity, and (5) share a bed with another person. When asked, none of the participants reported non-compliance.

### Method details

#### Dual-choice landing assay for relative attractiveness

The relative attractiveness of individual human participants to female *Ae. aegypti* was assessed in a dual-choice landing assay adapted from^80^ (Figure 1A). Twenty hours before the experiment, a group of 15 female mosquitoes (5 dpe) was transferred to the experimental cage (modified BugDorm cage, 30 cm × 30 cm × 30 cm) using a mouth aspirator, and provided only water *ad libitum*. Skin odor from each participant was collected directly prior to the experiment by rubbing three sandblasted glass tubes for 2 min each against the forearm of the participant. Three other sandblasted glass tubes were coated with 200 μL of a synthetic human odor blend^36^ (diluted 100×; Table S1) each. One glass tube of each kind was inserted into the two holders of the experimental cage and heated to 37°C using a water bath. During 3 min, the cumulative number of landings on each glass tube were recorded. The relative attractiveness index (RAI) for each of the three replicates per participant was calculated as the number of landings on the handled glass tube, divided by the total number of landings on both glass tubes. Experiments were conducted during the peak activity period of host seeking (zeitgeber time 10–14) for *Ae. aegypti*.^81^^,^^82^^,^^83^

#### Headspace odor collection from humans

Whole-body volatiles were collected according to Harraca et al.,^84^ using dynamic headspace extraction. Right after assessing their relative attractiveness (see paragraph above), undressed individuals were placed in customized bags made from roasting tubes (180 cm × 60 cm; Melitta, Minden, Germany; three roasting tubes per bag), with only their head protruding. The bag was fastened around the neck of the participant using tape, and the participant was covered with a blanket. Synthetic air (20.9% O_2_, 79.1% N_2_; Strandmöllen AB, Ljungby, Sweden) was passed into the bag through a port close to the shoulder at 3 L min^−1^, and six pumps (NMP830KNDC; KNF, Freiburg, Germany) extracted air at 0.4 L min^−1^ through twelve adsorbent columns arranged along the sides of the bag. Adsorbent columns were made from Teflon tubing (3 mm inner diameter) and contained 50 mg Porapak Q (80–100 mesh, Alltech Associates, USA) between glass wool plugs and Teflon stoppers. An empty previously unused participant bag with two adsorbent columns was used as a control. Headspace was sampled for 3 h during which time the participants could watch a movie, listen to podcasts or take a nap.

Volatiles adsorbed on Porapak Q were desorbed with 500 μL of pentane (Sigma-Aldrich) per column, and all samples obtained from an individual participant and the control were pooled, respectively. The samples were stored at −20°C until further use. For electrophysiological experiments and chemical analysis, a part of the pooled sample from the participant was carefully concentrated circa 25 times to a volume of approximately 25 μL, while the sample from the empty control bag was concentrated 10 times due to the lower initial elution volume. Columns and adapters were cleaned after elution with 1 mL of pentane each and the bag used for the participant discarded. The empty control bag was used as test bag for the next participant.

#### GC-EAD analysis of human headspace extracts

Antennal responses to the pooled headspace extracts of individual participants were analyzed by GC-EAD using an Agilent 6890N GC (Agilent Technologies, Santa Clara, USA). The GC was equipped with an HP-5MS fused silica column (30 m × 0.25 mm × 0.25 μm; Agilent Technologies), using hydrogen as the carrier gas at a constant flow rate of 45 cm s^−1^. Each sample (2 μL) was injected in splitless mode (30 s, injector temperature 225°C). The temperature program used was 40°C initial temperature, which was increased by 3°C·min^−1^ to 180°C, followed by a heating gradient of 15°C·min^−1^ to 280°C (2 min hold). The GC effluent was split 1:1 in a Gerstel 3D/2 low dead volume four-way cross (Gerstel, Mülheim, Germany) between the flame ionization detector and the EAD. The GC effluent for the EAD passed through a Gerstel ODP-2 transfer line, with the temperature matched to the GC oven, into a glass tube (30 cm, 8 mm diameter) and was mixed with charcoal filtered, humidified air (1.5 L min^−1^).

For EAD recordings, the excised head of a female *Ae. aegypti*, with the distal segment of the antennae cut off, was mounted between a ground electrode and a recording electrode, both filled with Beadle-Ephrussi ringer solution^85^ (Figure 2B). The preparation was placed 0.5 cm from the outlet of the glass tube. The EAD signals were filtered and pre-amplified (10x) using a high impedance DC amplifier interface box (IDAC-2; Syntech, Kirchgarten, Germany) connected to the recording electrode. At least three consistent GC-EAD recordings on different mosquito preparations were made for each of the 42 headspace extracts (Figure S1). The antennal responses to the human headspace samples were recorded and analyzed using GC-EAD 2011 (version 1.2.3, Syntech, Kirchzarten, Germany). Compounds that elicited a consistent EAD response in headspace extracts corresponding to at least three different participants were considered for further analysis. The few compounds from the synthetic human blend^36^ that did not meet this criterion were also added to the panel, as they were used for assessing the relative attractiveness of the participants. The identity of the panel of the 27 selected electrophysiologically-active compounds was confirmed by co-injecting synthetic standards (Table S2). Experiments were conducted during the peak activity period of host seeking (zeitgeber time 10–14) for *Ae. aegypti*.^81^^,^^82^^,^^83^

#### Identification and quantification of select compounds using GC-MS

Extracted volatile organic compounds were identified and quantified by combined GC and mass spectrometry (GC-MS), using both a DB-Wax column (60 m × 0.25 mm × 0.25 μm, Agilent Technologies) on an Agilent 7890B GC 5977A MS, and an HP-5MS UI column (60 m × 0.25 mm × 0.25 μm, Agilent Technologies) on an Agilent 6890 GC 5975 MS. For both instruments, 3 μL of the sample were injected in splitless mode (0.7 min, injector temperature 250°C), and helium was the carrier gas at a constant flow of 35 cm s^−1^. For runs on the DB-Wax column, the initial oven temperature was 40°C and increased by 5°C·min^−1^ to 230°C (10 min hold). For runs using the HP-5 column, the temperature program was the same as for the GC-EAD analysis. Total ion chromatograms were recorded by scanning from m/z 29 to m/z 400. Target compounds were tentatively identified according to their Kováts retention indices and mass spectra in comparison to the NIST20 library, and confirmed by co-injection of synthetic standards (Table S2). For quantification of the extracted compounds, external standards (10 ng μl^−1^) of the selected compounds were used. Total ion chromatogram peak areas were integrated using AMDIS 32 (National Institute of Standards and Technology, Gaithersburg, USA). Analysis settings (peak deconvolution) were adjusted to: adjacent peak subtraction – one, shape requirements – medium, medium resolution and low sensitivity. 2-Nonanol, a VOC of the synthetic human blend,^36^ was not detected in any of the headspace samples and was omitted from quantification.

The chirality of select compounds found in human headspace odor (α-pinene, β-pinene, limonene, linalool, menthol, 2-ethyl-1-hexanol) was determined using GC-MS and GC-EAD. For identification of the chiral compounds, a J&W Cyclodex-B column (60 m × 0.25 mm × 0.25 μm; Agilent Technologies) on an HP 6890 GC 5973 MS instrument (Hewlett-Packard, Palo Alto, USA) was used. Helium was the carrier gas at a flow rate of 32 cm s^−1^. The GC oven program was tailored to an optimal separation of peaks using synthetic standards (Table S4). Total ion chromatograms were recorded by scanning from m/z 29 to m/z 400. The elution order of the enantiomers was established using mixtures of different enantiomeric ratios. Then, headspace samples of five participants were pooled and concentrated to approximately 10 μL, of which either 2 μL or 3 μL were injected onto the GC-MS. When necessary for peak identification, synthetic standards were co-injected. The absolute configuration of 2-ethyl-1-hexanol was determined by partial resolution of the racemic compound with the use of Amano PS lipase.^86^^,^^87^ To record antennal responses to all enantiomers or the chiral compounds, the Cyclodex-B column described above was used for GC-EAD (see Table S4 for the custom temperature programs).

#### Assessment of synthetic blends using a dual-choice olfactometer

Based on the results of the chemical analysis, several synthetic odor blends were created, each including all 27 VOCs previously selected and identified by GC-EAD and GC-MS. For the “average” blend, their total amounts corresponded to the relative mean VOC abundance of all participants (stock solution: 2.69 mol ml^−1^; Table S5). For testing the role of (*R*)-1-octen-3-ol, the “average” blend was adjusted by adding (*R*)-1-octen-3-ol to match the relative abundance of this VOC, as found in highly attractive individuals (based on the panel of 27 VOCs, the human headspace samples contained 0.05% 1-octen-3-ol on average, but 0.3% in highly attractive individuals), and solvent to keep the concentration of the “average with ↑ (*R*)-1-octen-3-ol” stock solution at 2.69 mol mL^−1^ (Table S6). Furthermore, two additional blends were based on the relative mean VOC abundance of participants of low and very high attractiveness (Table S5). Additionally, the synthetic human odor blend^36^ used as a control for the assessment of the relative attractiveness of a participant was tested (Table S1).

The relative attractiveness of the synthetic blends to female *Ae. aegypti* was assessed in a dual-choice olfactometer^36^ (“Y-tube”; transparent Plexiglas; 120 cm × 10 cm inner diameter; Figure 4A). The olfactometer was placed on a surface with a dot-pattern for visual orientation of the mosquitoes and illuminated with red light (2–5 lux) from above. Charcoal-filtered and humidified air (26 ± 1°C, 75 ± 5% relative humidity) was pushed through the olfactometer at 30 cm s^−1^. Odor cues were presented in the two arms of the olfactometer, by pushing humidified air through two wash bottles (250 mL) containing one wick dispenser^88^ each. The wick dispensers ensured a constant release of the odor blend, and maintained blend composition, throughout the experiment.^88^ Before the experiment (2–3 h), female mosquitoes (5 dpe) were starved by changing the sucrose solution for distilled water, and then, 1–2 h before the experiment, transferred to release cages (6 cm × 10 cm inner diameter) in groups of 5 individuals. Each release cage was placed at the downwind end of the olfactometer and the door opened after 1 min of acclimatization. The choice of the mosquitoes for any of the two arms was recorded after 3–5 min. Trials in which no or just one mosquito responded by entering either of the arms of the olfactometer were excluded from further analysis. A minimum of 30 trials were run per experiment. For each trial, the preference index was calculated as the number of mosquitoes in the target arm, divided by the total number of mosquitoes entering both arms. As for the dual-choice landing assay, experiments were conducted during the peak activity period of host seeking (zeitgeber time 10–14^81^^,^^82^^,^^83^). In a first experiment, the optimal dilution of the “average” blend was established and further experiments were based on this concentration.

### Quantification and statistical analysis

#### Statistical analysis of the relative attractiveness and reported variables

Participants were divided into four groups, low, moderate, high, and very high, according to their mean attractiveness values by quartile normalization, *i.e*., the participants were divided into the four quartiles along the range of their mean RAI values. A Kruskal-Wallis rank-sum test using R^79^ (version 4.0.5) compared variations in the inter-individual mean relative attractiveness to mosquitoes, considering the following variables, if reported: pregnancy or menstrual cycle phase, ABO blood type, use of hormonal contraception, and age. Differences between the groups were tested in a pairwise manner by the Dunn’s test and adjusted for multiple comparisons using the Bonferroni-correction.

#### Statistical analysis of odor profiles

Data collected using the DB-Wax GC column was used for quantitative analysis. The abundance of the 27 selected VOCs extracted per participant was calculated as the difference between the amount of volatiles collected for the participant and the empty bag ×2.5 (there was a different concentration factor due to a lower initial elution volume of the control, to not lose the sample; see above). The level of attractiveness, along with pregnancy status or week of the menstrual cycle, the ABO blood type, and the use of hormonal contraception were considered as potential explanatory variables for the variation in individual odor profiles. Analyses of similarity (ANOSIM) were performed to test for differences in individual odor profiles using the R package “vegan”.^89^ Using the Gower dissimilarity measure as the distance metric, the ANOSIM test compared the mean of ranked dissimilarities between groups to the mean of ranked dissimilarities within groups. An ANOSIM statistic R value close to one suggests dissimilarity between groups, while an R value close to zero suggests an even distribution of high and low ranks within and between groups. A negative R value indicates that differences within groups are greater than between groups. ISA was used to identify specific VOCs that were consistently and significantly associated with particular groups or conditions, rather than comparing overall profiles. By focusing on individual compounds, ISA allowed to pinpoint biomarkers that characterize or distinguish groups. While both ISA and ANOSIM are statistical methods used to assess group differences, they differ in their approach and the insights they provide. While ISA identifies specific variables (*i.e*., VOCs) that are consistently and significantly associated with particular groups, this approach focuses on individual markers, allowing for the identification of compounds that characterize or distinguish groups. On the other hand, ANOSIM assesses whether there are significant differences in community composition between two or more groups, based on a ranked dissimilarity matrix. This complementary approach allowed for a more detailed understanding of the specific markers driving group differences. Participants with a high relative abundance of 1-octen-3-ol were determined using k-means clustering with k = 2 (Figure S4). In addition to comparing the entire profile of volatiles across the four groups of attractiveness levels, an ISA was performed, using the R package “indicspecies”,^90^ to identify specific compounds that may be consistently and significantly associated with certain groups.

#### Statistical analysis of the assessment of synthetic blends

Treatment effects were tested using a Kruskal-Wallis rank-sum test. *Post-hoc* comparisons of the preference index values against zero, thus testing for a non-random distribution of mosquitoes, were performed using one-sample two-tailed Wilcoxon tests with Bonferroni-correction for multiple comparisons.

## Supplemental information

Document S1. Figures S1–S4 and Tables S1–S6

Data S1. Informed consent form
