# Supplementary material for: Cyphers and cycles – A chemical basis of the differential attraction of mosquitoes to human odor
Source: iScience. 2026 Apr 2;29(5):115575. doi: 10.1016/j.isci.2026.115575 (PMC13127324; doi:10.1016/j.isci.2026.115575)
Supplement: Document S1. Figures S1–S4 and Tables S1–S6 [file mmc1.pdf]

iScience, Volume *29*

Supplemental information

Cyphers and cycles – A chemical basis of the differential attraction of mosquitoes to human odor

Annika Hinze, Anaïs Karine Tallon, Betelehem Wondwosen, Mengistu Dawit, Sharon Rose Hill, Björn Bohman, and Rickard Ignell

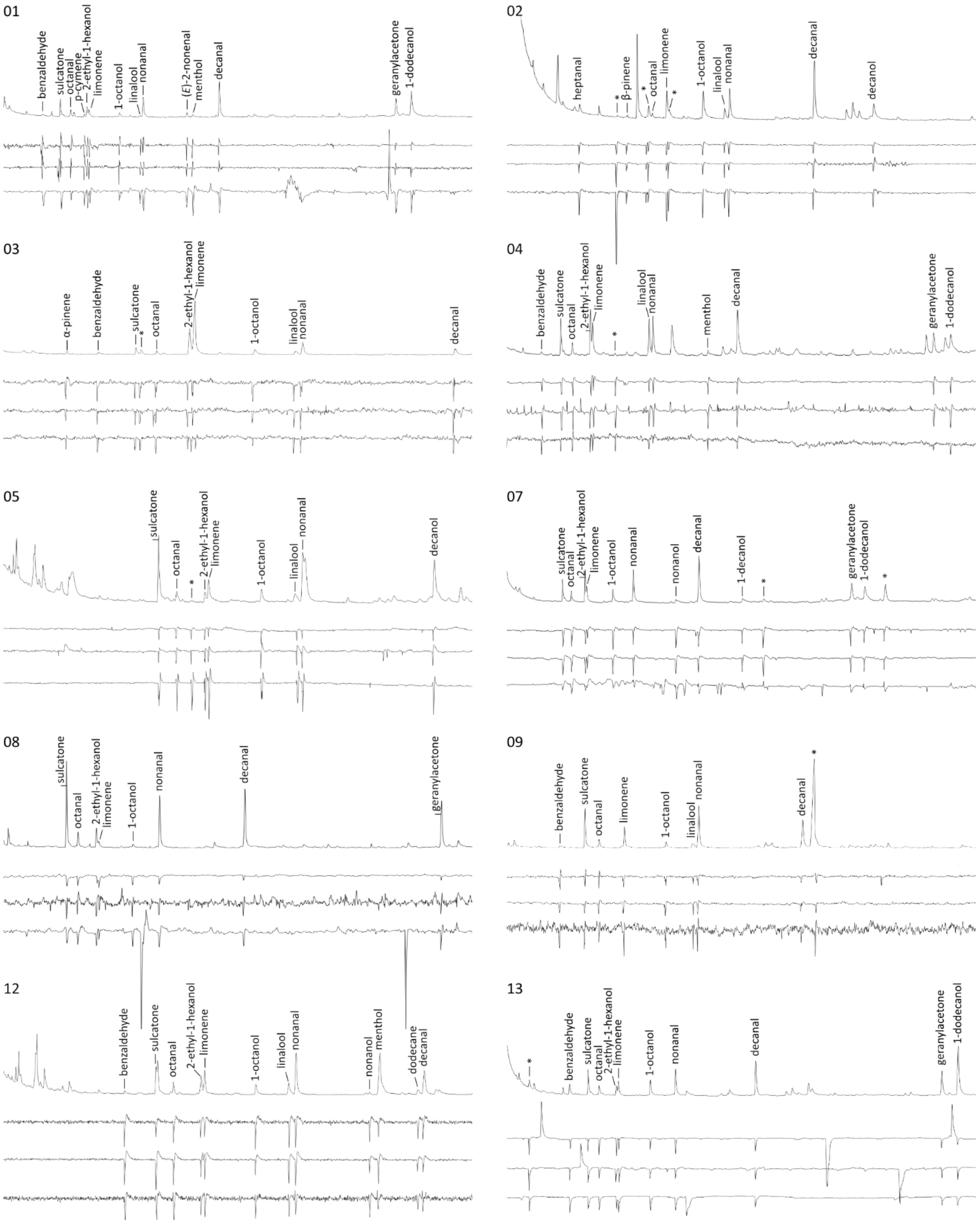

Figure S1A. Combined gas chromatography and electroantennographic detection (GC-EAD) analysis of antennal preparations of female *Ae. aegypti* to the headspace extracts of all human volunteers. Related to Figure 2C. Trace of the flame ionization detector (FID) above, EAD traces of three biological replicates below. Note the FID and EAD traces are not normalized comparing between volunteers. Numbers refer to the ID of the volunteer. All named compounds have been identified by co-injection with verified standards, whereas asterisks indicate unverified compounds.

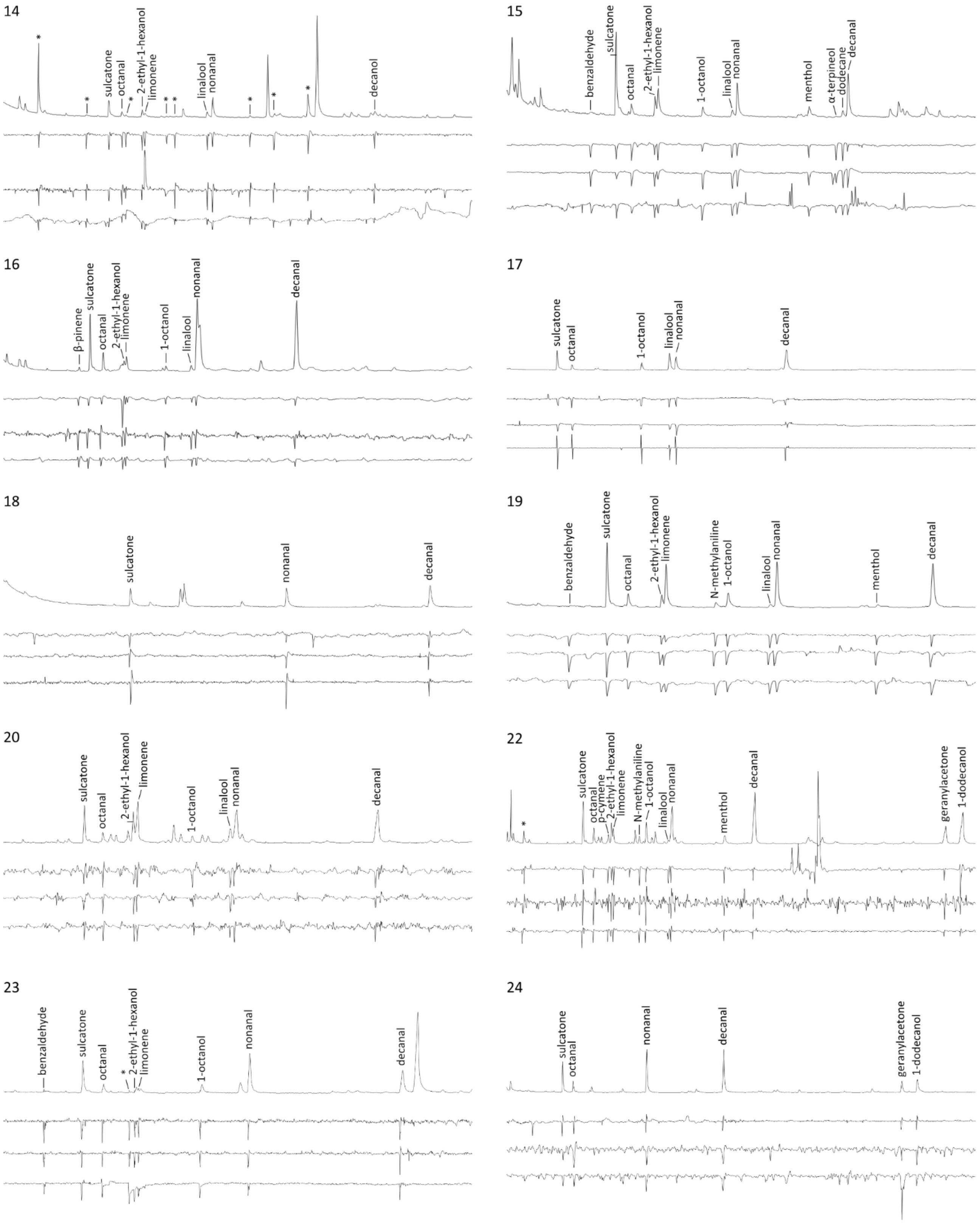

Figure S1B. Combined gas chromatography and electroantennographic detection (GC-EAD) analysis of antennal preparations of female *Ae. aegypti* to the headspace extracts of all human volunteers. Related to Figure 2C. Trace of the flame ionization detector (FID) above, EAD traces of three biological replicates below. Note the FID and EAD traces are not normalized comparing between volunteers. Numbers refer to the ID of the volunteer. All named compounds have been identified by co-injection with verified standards, whereas asterisks indicate unverified compounds.

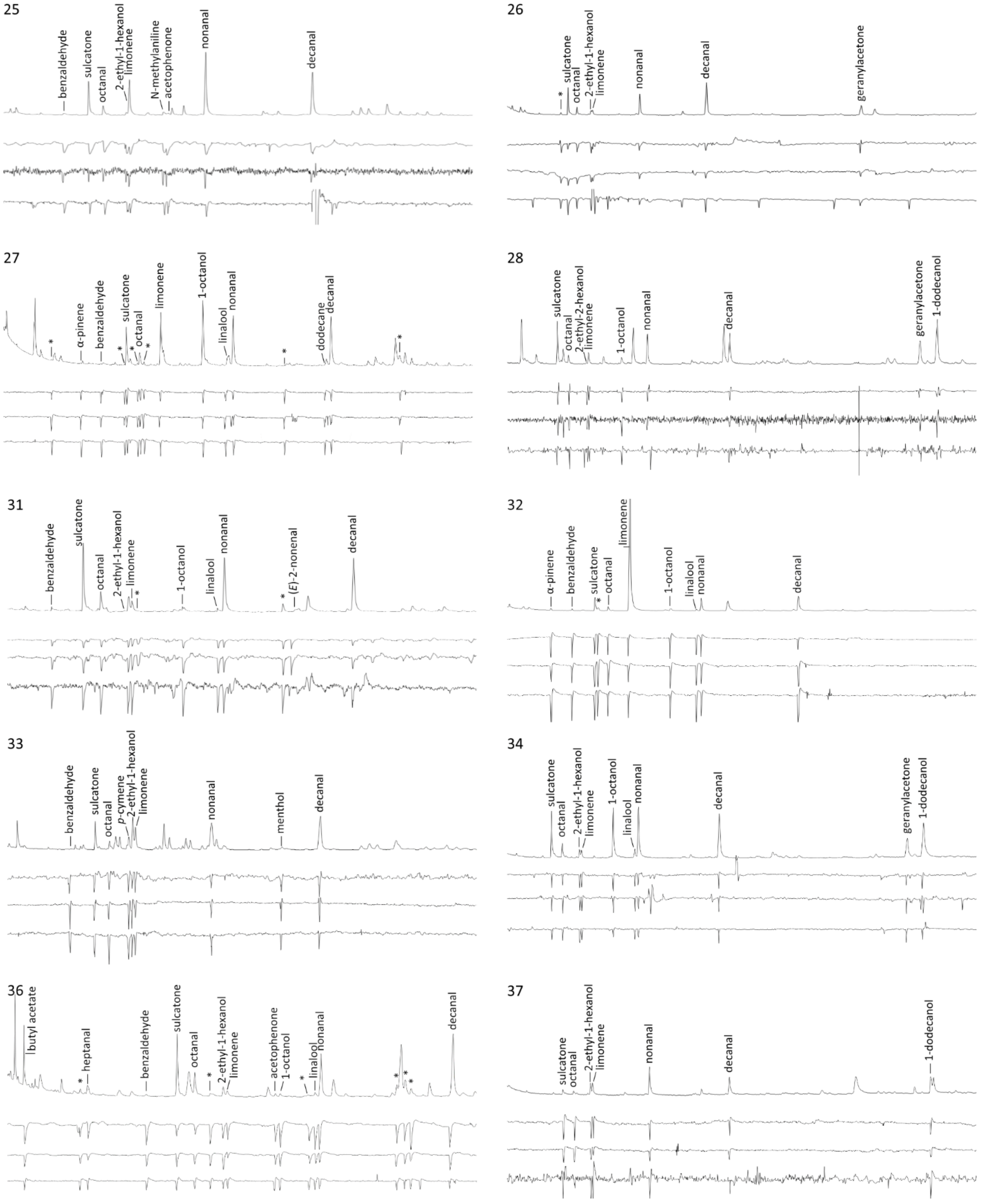

Figure S1C. Combined gas chromatography and electroantennographic detection (GC-EAD) analysis of antennal preparations of female *Ae. aegypti* to the headspace extracts of all human volunteers. Related to Figure 2C. Trace of the flame ionization detector (FID) above, EAD traces of three biological replicates below. Note the FID and EAD traces are not normalized comparing between volunteers. Numbers refer to the ID of the volunteer. All named compounds have been identified by co-injection with verified standards, whereas asterisks indicate unverified compounds.

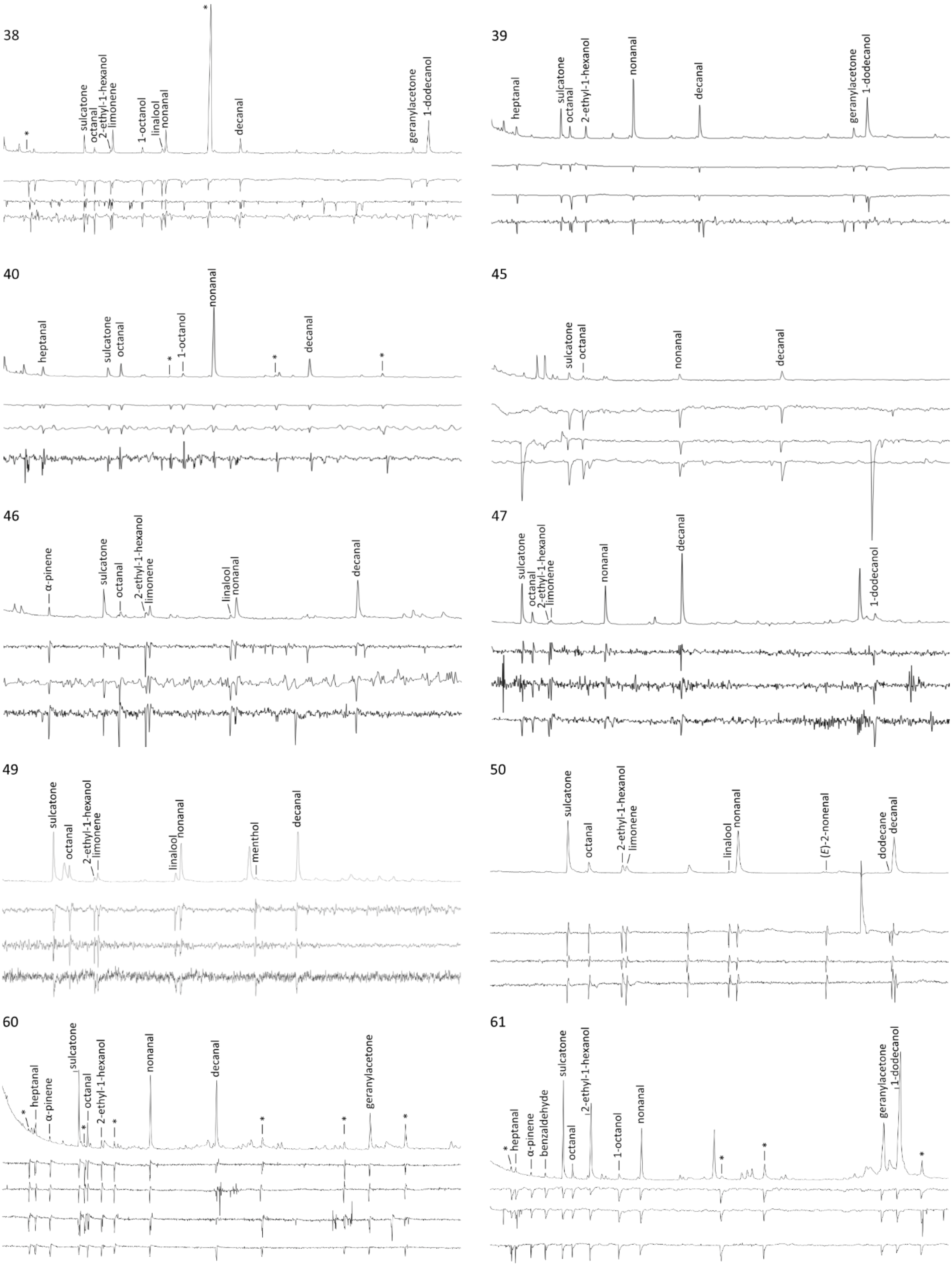

Figure S1D. Combined gas chromatography and electroantennographic detection (GC-EAD) analysis of antennal preparations of female *Ae. aegypti* to the headspace extracts of all human volunteers. Related to Figure 2C. Trace of the flame ionization detector (FID) above, EAD traces of three biological replicates below. Note the FID and EAD traces are not normalized comparing between volunteers. Numbers refer to the ID of the volunteer. All named compounds have been identified by co-injection with verified standards, whereas asterisks indicate unverified compounds.

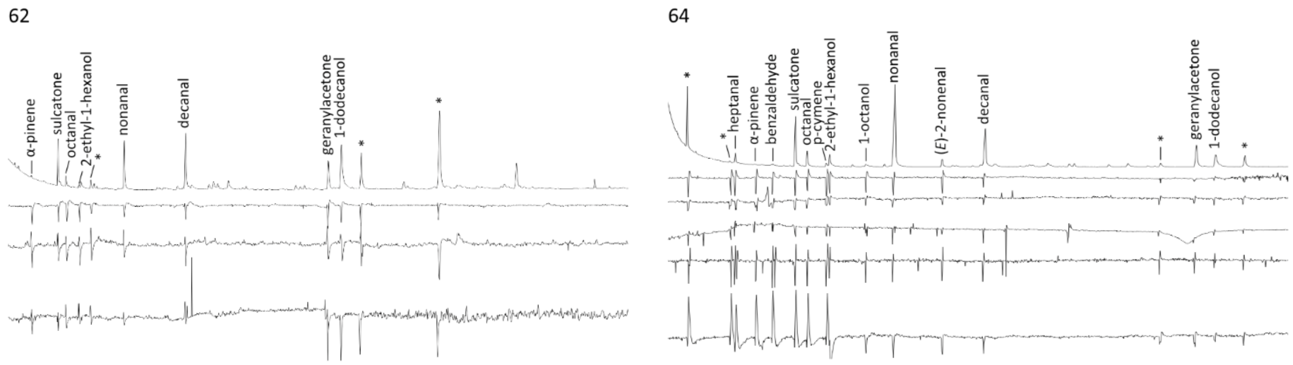

Figure S1E. Combined gas chromatography and electroantennographic detection (GC-EAD) analysis of antennal preparations of female *Ae. aegypti* to the headspace extracts of all human volunteers. Related to Figure 2C. Trace of the flame ionization detector (FID) above, EAD traces of three biological replicates below. Note the FID and EAD traces are not normalized comparing between volunteers. Numbers refer to the ID of the volunteer. All named compounds have been identified by co-injection with verified standards, whereas asterisks indicate unverified compounds.

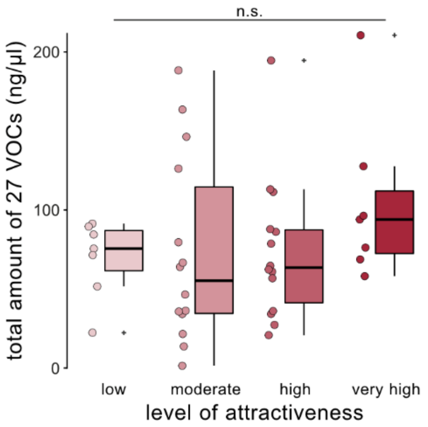

Figure S2. Total amount of the panel of 27 volatile organic compounds (eliciting consistent electrophysiological antennal responses in *Aedes aegypti* to the headspace extracts of at least three volunteers and/or the VOCs from the behavioral screening) extracted per volunteer, grouped by the level of attractiveness. Boxes represent upper and lower quartiles, whiskers denote 1.5 times interquartile distance, crosses outliers, and black horizontal lines the median. There was no significant effect of VOC abundance on the level of attractiveness (Kruskal-Wallis, $p = 0.32$).

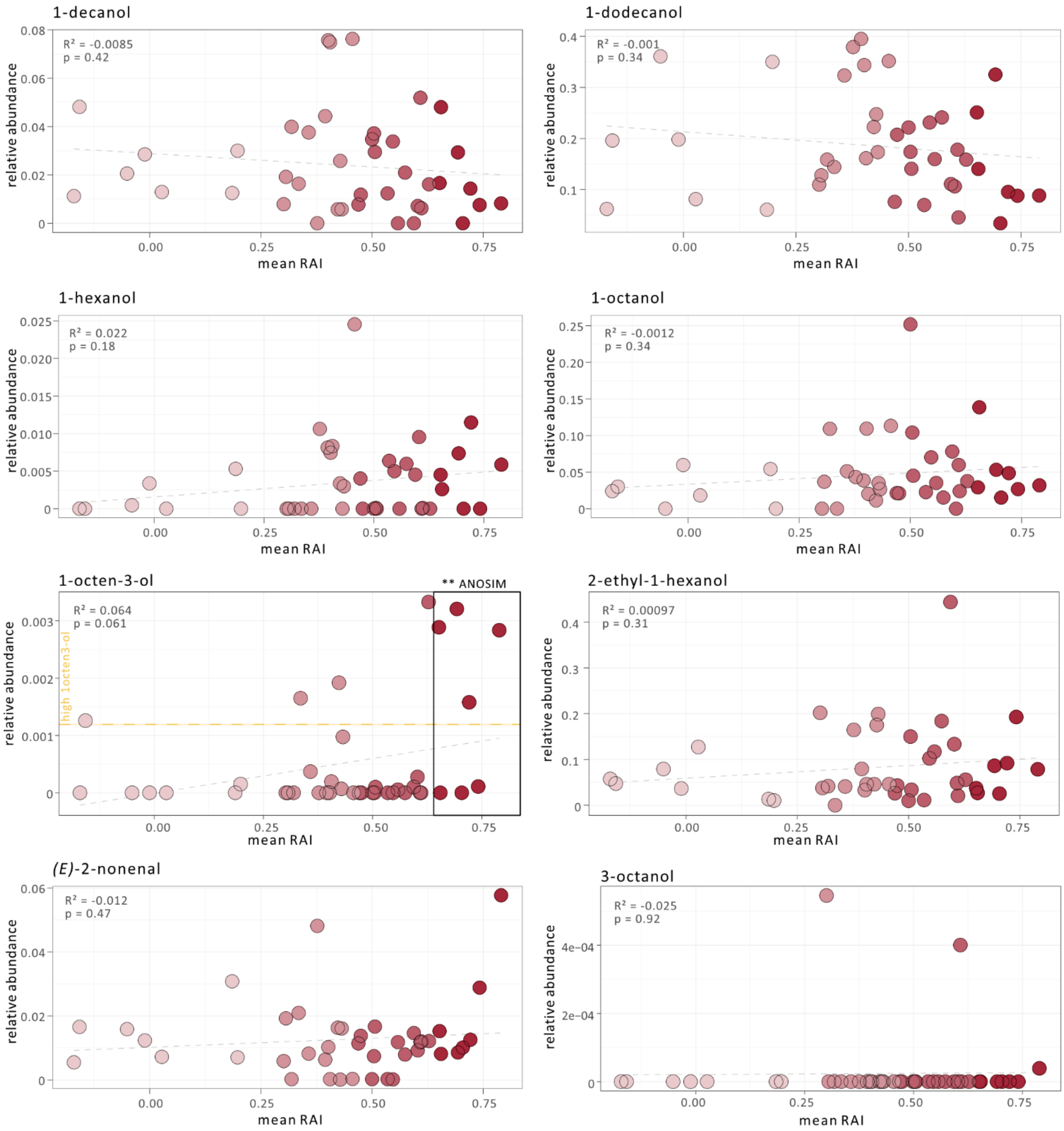

Figure S3A. Relative abundance index (RAI) of volatile organic compounds (in alphabetical order), compound classes (aldehydes, terpenoids), VOC proportions (decanal to 1-hexanol) and major human sebum-derived compounds (sulcatone, geranylacetone, and decanal) in relation to the individual mean relative attractiveness. Related to Figure 3. The attractiveness level is denoted by the lightness of red, from low (light red) to very high (dark red). For ease of interpretation, the linear model is depicted as a line within the graphs, although this mode of analysis is not well-suited (uniformly low R-squared values indicate low linear association). The black box highlights VOCs with significantly higher similarity within (ISA) rather than between levels of attractiveness (ANOSIM): 1-octen-3-ol. "High 1-octen-3-ol" was defined by k-means clustering of the relative abundance with k = 2, the yellow line denotes the midpoint between the cluster means: 0.0012.

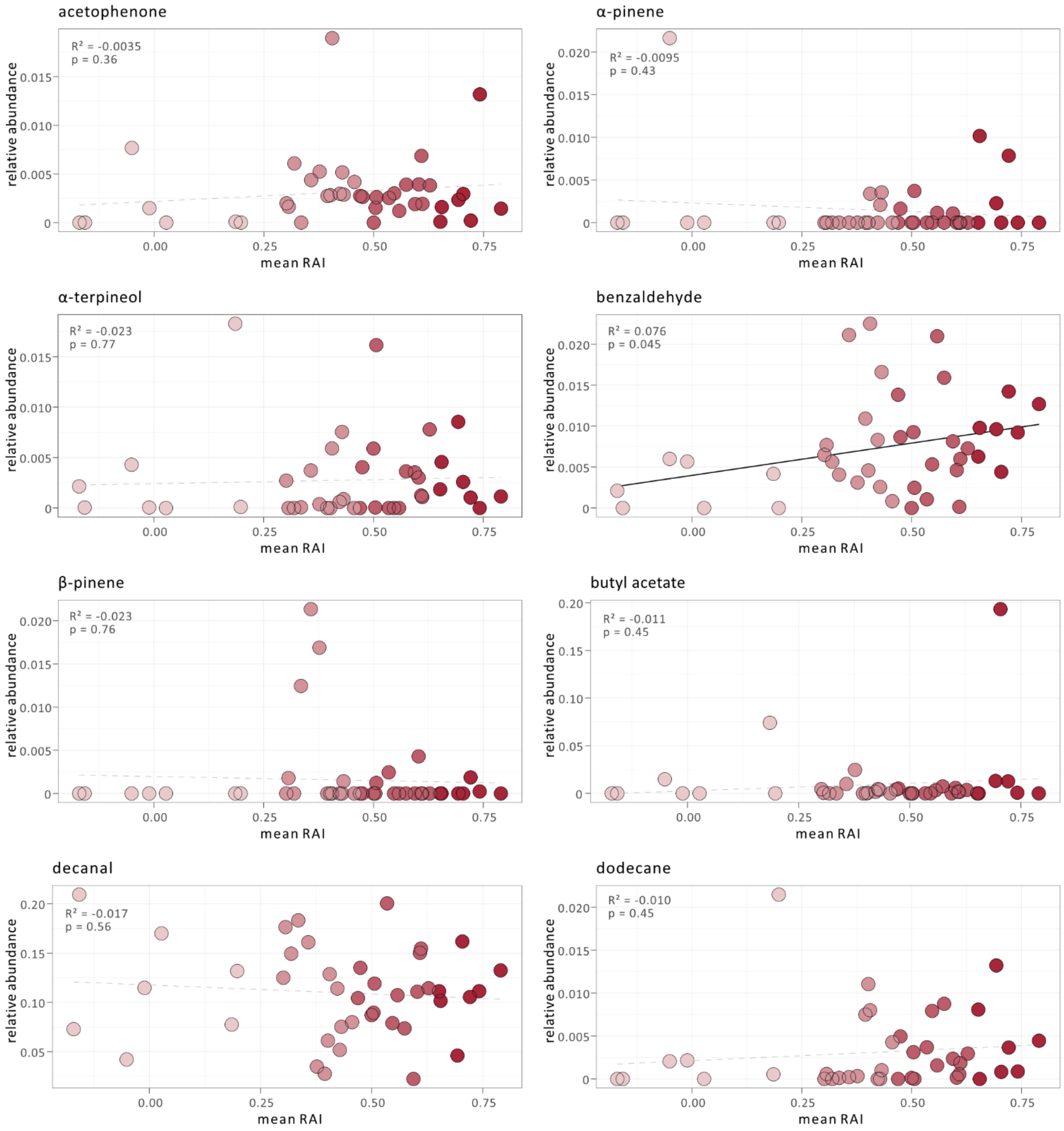

Figure S3B. Relative abundance index (RAI) of volatile organic compounds (in alphabetical order), compound classes (aldehydes, terpenoids), VOC proportions (decanal to 1-hexanol) and major human sebum-derived compounds (sulcatone, geranylacetone, and decanal) in relation to the individual mean relative attractiveness. Related to Figure 3. The attractiveness level is denoted by the lightness of red, from low (light red) to very high (dark red). For ease of interpretation, the linear model is depicted as a line within the graphs, although this mode of analysis is not well-suited (uniformly low R-squared values indicate low linear association).

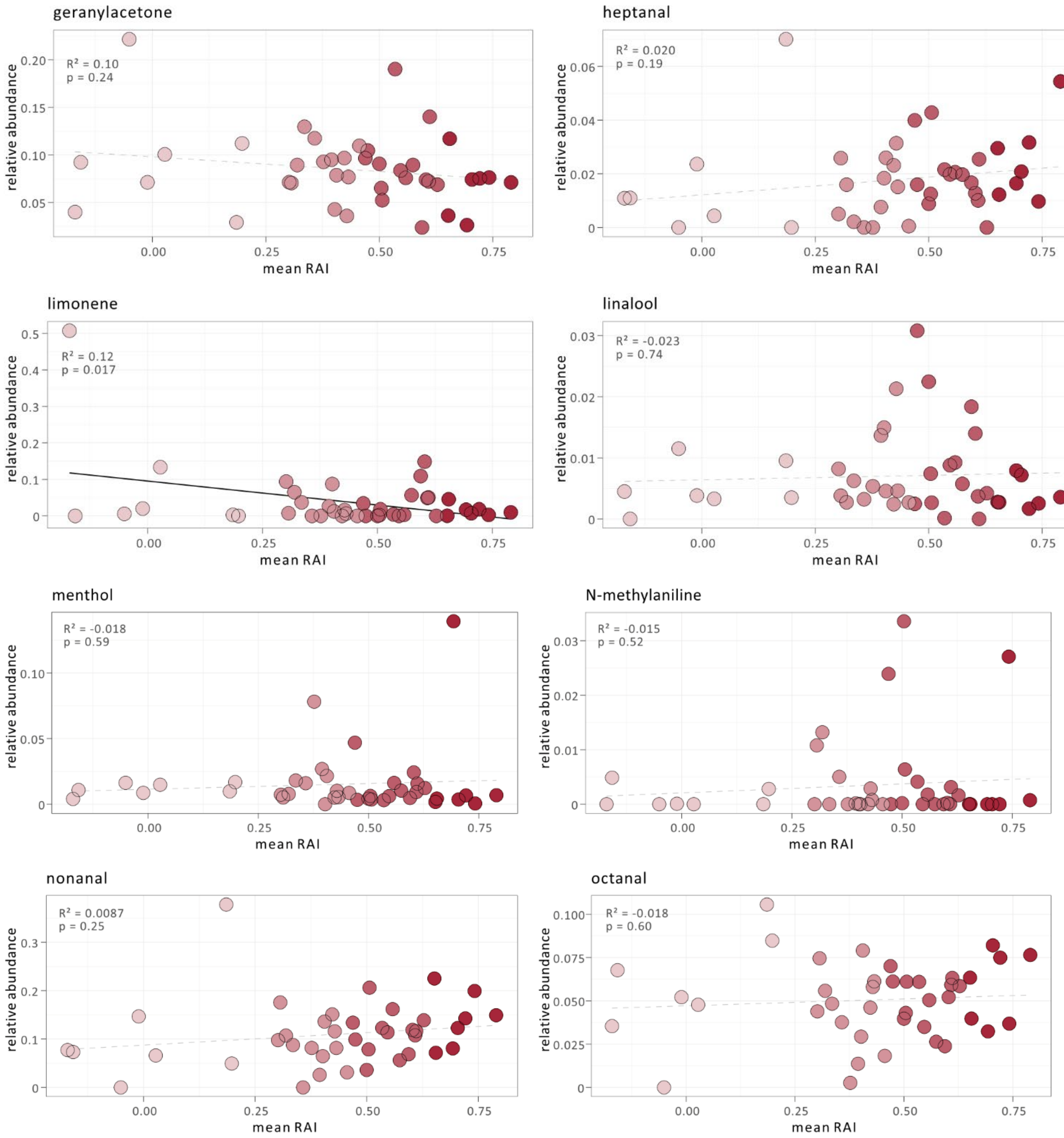

Figure S3C. Relative abundance index (RAI) of volatile organic compounds (in alphabetical order), compound classes (aldehydes, terpenoids), VOC proportions (decanal to 1-hexanol) and major human sebum-derived compounds (sulcatone, geranylacetone, and decanal) in relation to the individual mean relative attractiveness. Related to Figure 3. The attractiveness level is denoted by the lightness of red, from low (light red) to very high (dark red). For ease of interpretation, the linear model is depicted as a line within the graphs, although this mode of analysis is not well-suited (uniformly low R-squared values indicate low linear association).

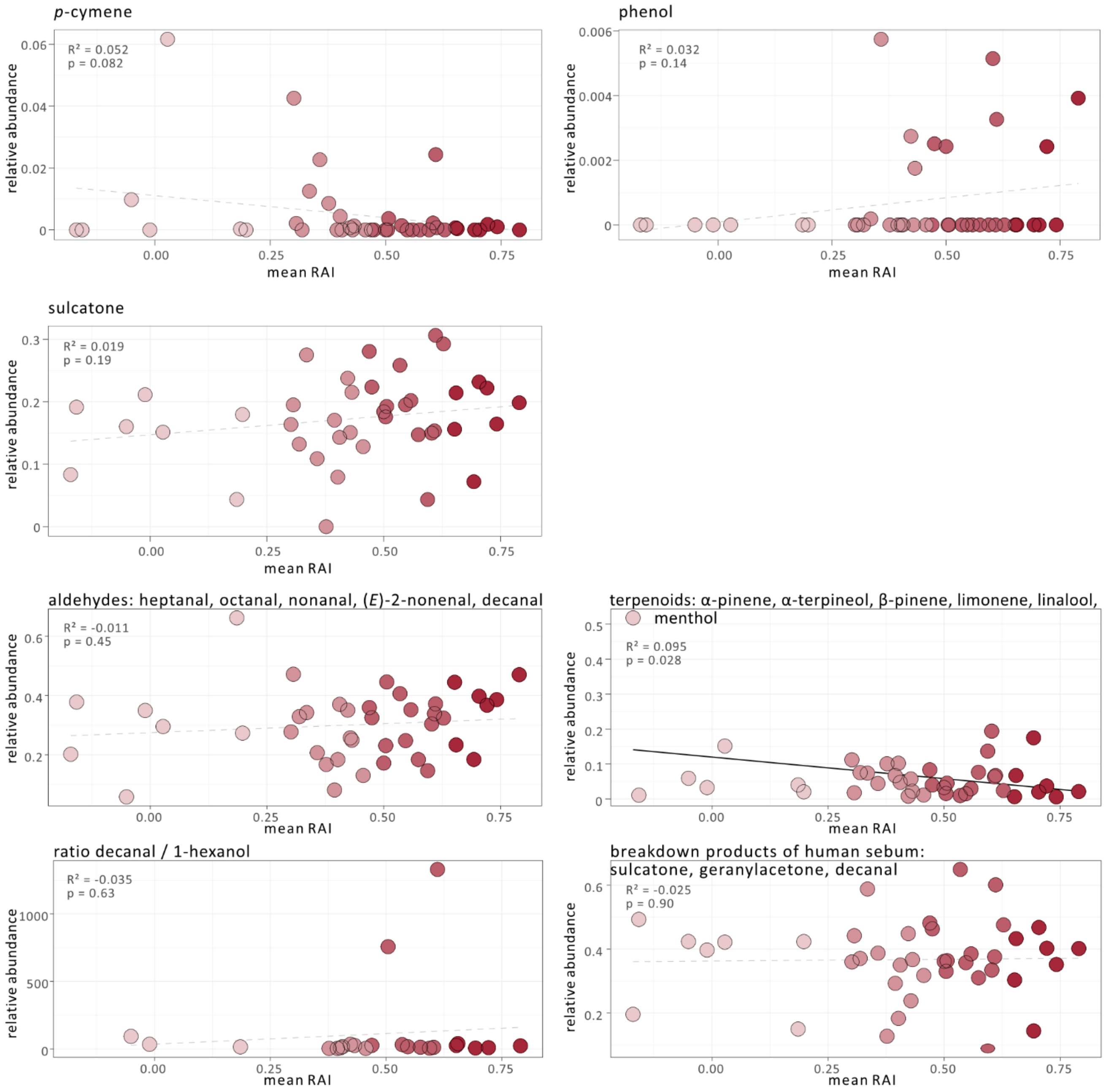

Figure S3D. Relative abundance index (RAI) of volatile organic compounds (in alphabetical order), compound classes (aldehydes, terpenoids), VOC proportions (decanal to 1-hexanol) and major human sebum-derived compounds (sulcatone, geranylacetone, and decanal) in relation to the individual mean relative attractiveness. Related to Figure 3. The attractiveness level is denoted by the lightness of red, from low (light red) to very high (dark red). For ease of interpretation, the linear model is depicted as a line within the graphs, although this mode of analysis is not well-suited (uniformly low R-squared values indicate low linear association).

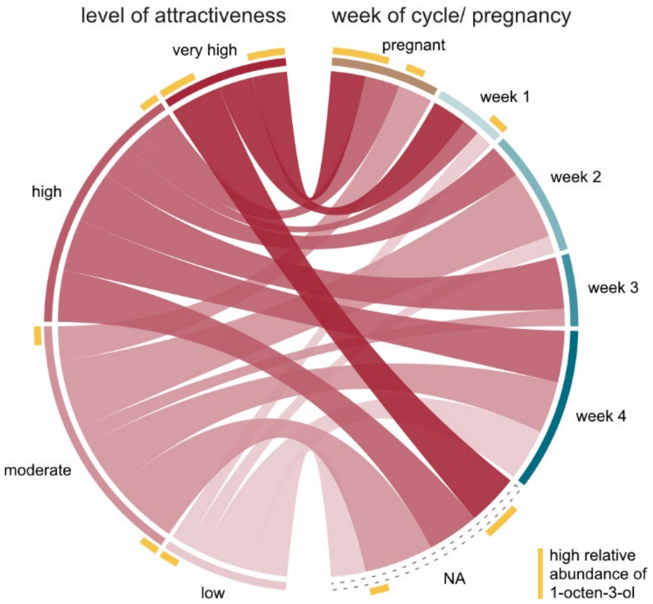

Figure S4. Chord diagram of the relation between the level of attractiveness and the week of the menstrual cycle or pregnancy. Related to Figures 3 and S3 (panel "1-octen-3-ol"). Node colors correspond to the level of attractiveness (left) or the week of the menstrual cycle or pregnancy (right), ribbon colors to the level of attractiveness. Ribbon width corresponds to the number of participants. NA refers to participants that did not (or could not) share information about the week of their menstrual cycle or pregnancy. Participants with a high relative abundance of 1-octen-3-ol (> 0.12%; see Figure S3; groups defined by k-means clustering with k = 2) in their headspace odor are highlighted in yellow.

Table S1. Content of the synthetic human odor blend [S1] used in the dual-choice landing assay. Related to Figures 1 and 4, as well as Table S2.

compound	CAS	volume (µl)	specification
1-hexanol	111-27-3	5.7	see Table S2
(*R*)-1-octen-3-ol	3687-48-7	1.0	
2-nonanol	628-99-9	1.1	
3-octanol	589-98-0	18	
acetophenone	98-86-2	0.4	
α-terpineol	10482-56-1	3.3	
benzaldehyde	100-52-7	4.4	
butyl acetate	123-86-4	4.8	
decanal	112-31-2	115	
(+)-limonene	5989-27-5	150	
linalool	78-70-6	5.7	
nonanal	124-19-6	76	
octanal	124-13-0	7.7	
phenol	108-95-2	1.0	
sulcatone	110-93-0	51	
pentane	109-66-0	611	
		1000	

Table S2. Neat compounds used for the synthetic blends (Tables S1, S5, S6) and the chemical analyses.

compound	IUPAC name	CAS	purity (%)	company
1-decanol	decan-1-ol	112-30-1	≥99	Fluka
1-dodecanol	dodecan-1-ol	112-53-8	≥99	Fluka
1-hexanol	hexan-1-ol	111-27-3	98	Sigma-Aldrich
1-octanol	octan-1-ol	111-87-5	≥99.5	Fluka
(*R*)-1-octen-3-ol	(3*R*)-oct-1-en-3-ol	3687-48-7	98	Sigma-Aldrich
2-ethyl-1-hexanol	2-ethylhexan-1-ol	104-76-7	≥99	Fluka
2-nonanol	nonan-2-ol	628-99-9	99	Sigma-Aldrich
(*E*)-2-nonenal	(*E*)-non-2-enal	18829-56-6	97	Sigma-Aldrich
3-octanol	octan-3-ol	589-98-0	≥95	Fluka
acetophenone	1-phenylethanone	98-86-2	99	Acros Organics

(+)-α-pinene	(1*R*,5*R*)-4,6,6-trimethylbicyclo[3.1.1]hept-3-ene	7785-70-8	98	Sigma-Aldrich
(-)-α-pinene	(1*S*,5*S*)-2,6,6-trimethylbicyclo[3.1.1]hept-2-ene	7785-26-4	99	Sigma-Aldrich
(+)/(-)-α-pinene	2,6,6-trimethylbicyclo[3.1.1]hept-2-ene	80-56-8	98	Sigma-Aldrich
(-)-α-terpineol	2-[(1*S*)-4-methylcyclohex-3-en-1-yl]propan-2-ol	10482-56-1	>98	Merck
benzaldehyde	benzaldehyde	100-52-7	≥95	Sigma-Aldrich
(+)-β-pinene	(1*R*,5*R*)-6,6-dimethyl-2-methylidenebicyclo[3.1.1]heptane	19902-08-0	≥95	Supelco
(-)-β-pinene	(1*S*,5*S*)-6,6-dimethyl-2-methylidenebicyclo[3.1.1]heptane	18172-67-3	99	Sigma-Aldrich
butyl acetate	butyl acetate	123-86-4	≥99.5	Sigma-Aldrich
decanal	decanal	112-31-2	≥98	Sigma-Aldrich
dodecane	dodecane	112-40-3	≥95	Fluka
geranylacetone	(5*E*)-6,10-dimethylundeca-5,9-dien-2-one	3796-70-1	65*	Alfa Aesar
			≥97	SAFC
heptanal	heptanal	111-71-7	95	Sigma-Aldrich
			≥92	SAFC
hexane	hexane	110-54-3	≥99.5	Merck
(+)-isomenthol	(1*S*,2*R*,5*R*)-5-methyl-2-propan-2-ylcyclohexan-1-ol	23283-97-8	≥95	PhytoLab
(+)-limonene	(4*R*)-1-methyl-4-prop-1-en-2-ylcyclohexene	5989-27-5	97	Sigma-Aldrich
(-)-limonene	(4*S*)-1-methyl-4-prop-1-en-2-ylcyclohexene	5989-54-8	96	Sigma-Aldrich
(+)/(-)-linalool	3,7-dimethylocta-1,6-dien-3-ol	78-70-6	97	Sigma-Aldrich
(-)-linalool	(3*R*)-3,7-dimethylocta-1,6-dien-3-ol	126-91-0	97	Fluka
(+)-menthol	(1*S*,2*R*,5*S*)-5-methyl-2-propan-2-ylcyclohexan-1-ol	15356-60-2	99	Sigma-Aldrich
(-)-menthol	(1*R*,2*S*,5*R*)-5-methyl-2-propan-2-ylcyclohexan-1-ol	2216-51-5	99.7	Acros Organics
m-cymene	1-methyl-3-propan-2-ylbenzene	535-77-3	≥95	Supelco
nerylacetone	(5*Z*)-6,10-dimethylundeca-5,9-dien-2-one	3879-26-3	≥98	Fluka
N-methylaniline	*N*-methylaniline	100-61-8	99	AK Scientific
nonanal	nonanal	124-19-6	≥95	Fluka
octanal	octanal	124-13-0	≥98	Fluka
o-cymene	1-methyl-2-propan-2-ylbenzene	527-84-4	98	Sigma-Aldrich
p-cymene	1-methyl-4-propan-2-ylbenzene	99-87-6	99	Sigma-Aldrich
pentane	pentane	109-66-0	≥99.5	Supelco
phenol	phenol	108-95-2	>99.5	Sigma-Aldrich

sulcatone	6-methylhept-5-en-2-one	110-93-0	99	Sigma-Aldrich

*contains 35% nerylacetone.

Table S3. Enantiomeric selectivity of female *Aedes aegypti* antennal preparations to select chiral compounds (GC-EAD) and occurrence in pooled human headspace extracts (GC-MS). Related to Figure 2E and Table S4.

compound	GC-EAD active	human headspace
(+)-α-pinene	+	(-) > (+)
(-)-α-pinene	-	
(+)-β-pinene	-	(+) ≥ (-)
(-)-β-pinene	+	
(+)-2-ethyl-1-hexanol	+	(+) ≥ (-)
(-)-2-ethyl-1-hexanol	-	
(+)-limonene	+	(+) >>> (-)
(-)-limonene	-	
(+)-linalool	+	(+) > (-)
(-)-linalool	-	
(+)-menthol	-	
(-)-menthol	+	(-)
(+)-isomenthol	-	

Table S4. Temperature programs of the GC oven used for the separation of chiral compounds. Related to Table S3.

enantiomers	initial temp.	ramp	end temp.	elution order
(+)-α-pinene, (-)-α-pinene	50 °C (2 min hold)	2 °C·min^{-1} to 225 °C	225 °C (5 min hold)	1: (-)-α-pinene, 2: (+)-α-pinene
(+)-β-pinene, (-)-β-pinene	50 °C (2 min hold)	5 °C·min^{-1} to 75 °C, 1 °C·min^{-1} to 105 °C, 5 °C·min^{-1} to 230 °C	230 °C (5 min hold)	1: (+)-β-pinene, 2: (-)-β-pinene
(+)-2-ethyl-1-hexanol, (-)-2-ethyl-1-hexanol	55 °C (2 min hold)	5 °C·min^{-1} to 100 °C, 1.5 °C·min^{-1} to 125 °C, 5 °C·min^{-1} to 230 °C	230 °C (5 min hold)	1: (-)-2-ethyl-1-hexanol, 2: (+)-2-ethyl-1-hexanol
(+)-limonene, (-)-limonene	50 °C (2 min hold)	5 °C·min^{-1} to 80 °C, 1 °C·min^{-1} to 100 °C, 5 °C·min^{-1} to 230 °C	230 °C (5 min hold)	1: (-)-limonene, 2: (+)-limonene
(+)-linalool, (-)-linalool	55 °C (2 min hold)	5 °C·min^{-1} to 110 °C, 1 °C·min^{-1} to 150 °C, 5 °C·min^{-1} to 225 °C	225 °C (5 min hold)	1: (-)-linalool, 2: (+)-linalool
(+)-menthol, (-)-menthol, (+)-isomenthol	50 °C (2 min hold)	5 °C·min^{-1} to 120 °C, 1 °C·min^{-1} to 150 °C, 5 °C·min^{-1} to 225 °C	225 °C (5 min hold)	1: (+)-menthol, 2: (-)-menthol, 3: (+)-isomenthol
mix 1: (+)-β-pinene, (-)-β-pinene, (+)-linalool, (-)-linalool, (+)-menthol, (-)-menthol, (+)-isomenthol	50 °C (2 min hold)	5 °C·min^{-1} to 83 °C, 1 °C·min^{-1} to 93 °C, 5 °C·min^{-1} to 103 °C, 1 °C·min^{-1} to 125 °C, 7 °C·min^{-1} to 227 °C	225 °C (5 min hold)	/
mix 2: (+)-α-pinene, (-)-α-pinene, (+)-limonene, (-)-limonene, (-)-2-ethyl-1-hexanol, (+)-2-ethyl-1-hexanol	50 °C (2 min hold)	2 °C·min^{-1} to 84 °C, 1 °C·min^{-1} to 92 °C, 5 °C·min^{-1} to 105 °C, 1.5 °C·min^{-1} to 118 °C, 7 °C·min^{-1} to 227 °C	225 °C (5 min hold)	/

Table S5. Content of the synthetic odor blends based on different levels of attractiveness. Related to Figure 4.

compound	CAS	"average"	"low"	"very high"	Omondi *et al.* [S1]	specification
		volume (µl)				
1-decanol	112-30-1	65	24	18.2	see Table S1	see Table S2
1-dodecanol	112-53-8	588	226	176		
1-octanol	111-87-5	97	23	42		
1-hexanol	111-27-3	5.7	0.9	3.1		
(*R*)-1-octen-3-ol[a]	3687-48-7	1.04[a]	0.2[a]	1.3[a]		
(-)/(+)-2-ethyl-1-hexanol	104-76-7	170	45	65		
(*E*)-2-nonenal	18829-56-6	28	12.0	17.8		
3-octanol[a]	589-98-0	0.05[a]	0[a]	0.005[a]		
acetophenone	98-86-2	4.8	0.8	2.0		
(+)/(-)-α-pinene	80-56-8	3.0	2.6	2.5		
α-terpineol	10482-56-1	6.0	3.2	2.5		
benzaldehyde	100-52-7	9.9	1.4	5.2		
(-)-β-pinene	18172-67-3	3.9	0	0.3		
butyl acetate	123-86-4	16.5	9.0	22.3		
decanal	112-31-2	270	119	112		
dodecane	112-40-3	13.9	4.6	5.5		
geranylacetone	3796-70-1	252	115	82		
heptanal	111-71-7	33	12.9	18.8		
(+)-limonene	5989-27-5	83	83	12.4		
(+)/(-)-linalool	78-70-6	16.4	4.9	3.9		
(-)-menthol	2216-51-5	37	10.7	21.5		
N-methylaniline	100-61-8	5.2	0.6	2.3		
nonanal	124-19-6	248	105	131		
octanal	124-13-0	104	47	49		
p-cymene	99-87-6	10.2	8.6	0.5		
phenol	108-95-2	2.9	0	0.4		
sulcatone	110-93-0	335	116	143		
hexane	110-54-3	2576	1023	1047		
		5000	2000	2000		

[a]These compounds were diluted 1:10 and 1:100 in hexane, respectively, before addition to the blend. The volumes given are the calculated volumes prior to dilution.

Table S6. Content of the synthetic odor blend based on the "average" blend (see Table S5), with the abundance of (*R*)-1-octen-3-ol increased to match the mean relative abundance found in the headspace of highly attractive individuals. Related to Figure 4 and Table S5.

compound	CAS	volume (µl)	specification
"average" blend (see Table S5)		1000	
(*R*)-1-octen-3-ol[a]	3391-86-4	0.88[a]	see Table S2
hexane	110-54-3	1.5	

[a]This compound was diluted 1:10 in hexane before addition to the blend. The volume given is the calculated volume prior to dilution.

Supplemental References

[S1] Omondi, A.B., Ghaninia, M., Dawit, M., Svensson, T., and Ignell, R. (2019). Age-dependent regulation of host seeking in Anopheles coluzzii. Sci. Rep. 9, 9699. 10.1038/s41598-019-46220-w.
