# Supplementary material for: Cyphers and cycles – A chemical basis of the differential attraction of mosquitoes to human odor
Source: iScience. 2026 Apr 2;29(5):115575. doi: 10.1016/j.isci.2026.115575 (PMC13127324; doi:10.1016/j.isci.2026.115575)
Supplement: Data S1. Informed consent form [file mmc2.zip › dataS1_informedconsentform.pdf]

Human Odour Collection Study

Informed Consent Form

This Informed Consent Form has two parts:

1. information sheet (see separate sheet)
2. certificate of consent

You will be given a copy of the full Informed Consent Form.

If you have any further questions, please contact the reasearchers conducting this study:

Annika Hinze / Anaïs Tallon
Disease Vector Group, Department of Plant Protection, SLU Alnarp
PO Box 102
Sundsvägen 14
230 53 Alnarp, Sweden

annika.hinze@slu.se / anais.tallon@slu.se

CERTIFICATE OF CONSENT

Statement by the volunteer

I have read the foregoing information. I have had the opportunity to ask questions about it and any questions I have been asked have been answered to my satisfaction. I consent voluntarily to be a participant in this study.

Name:

Date and signature:

Statement by the researcher

I confirm that the participant was given an opportunity to ask questions about the study, and all the questions asked by the participant have been answered correctly and to the best of my ability. I confirm that the volunteer has not been coerced into giving consent, and the consent has been given freely and voluntarily.

A copy of this form has been provided to the participant.

Name:

Date and signature:

Human Odour Collection Study

Information for Volunteers

About the Study

With this study, we would like to investigate why some individuals are more attractive to mosquitoes than others, focusing on individual human odour profiles and connecting it to a person's ABO blood type and physiological state, such as pregnancy or the week of the menstrual cycle. To do so, we will rate your body odour according to the attractiveness to mosquitoes and collect your whole body odour. We would like you to follow certain rules regarding food and the use of cosmetics the 24 h before the odour collection, since your diet and life style has an impact on your body odour. More information about the individual parts and the handling of personal data is given below. Please also not the "useful tips" at the bottom. As your participation is fully voluntarily, you have the right to retract your participation at any time.

If you have any further questions, please do not hesitate to contact me!

Annika Hinze: annika.hinze@slu.se

24 h before odour collection
follow life style regulations (see below)

→

day of experiments
attractiveness test,
odour collection

Life style regulations 24 h before odour collection

Before explaining the procedure of the attractiveness test and odour collection, please notice the instructions on **life style regulations**. It is crucial for the success of this study that you follow the instructions carefully. The goal is to minimise any odour contamination by, *e.g.*, the use of perfumes and cosmetics, or your diet.

24 h before odour collection:

(1) Do not use perfumes, deodorants, conventional shower products or any other cosmetics that contain perfumes, as well as any products that can interfere with body odour production such as antiperspirants. We will provide you with non-perfumed soap to use from 24 h before odour collection.
(2) Do not eat any garlic, onion, chilli, pepper (spicy food in general), blue cheese, vinegar, cabbage, radish, fermented milk products, or marinated fish.
(3) Do not engage in any intense physical activity.
(4) Do not sleep in bed of partners or anyone else the night before odour collection.

Do not hesitate to contact us with any question!

Attractiveness Test and Odour Collection

About the Attractiveness Test

Before odour collection, we will test the relative attractiveness of your body odour to mosquitoes. This test does not involve any direct contact with mosquitoes. We will ask you to rub three glass rods on your forearm and by doing so transfer your odour to them. How much mosquitoes like your odour will then be tested with these glass rods.

About the Odour Collection

For odour collection from plants and other small objects, it is common practice to place them in plastic oven bags and collect their odour by pumping the air from the bag through attached columns (a kind of filter that traps the odours). The basic principle to collect odour from humans is the same. You will need to stay in a human-sized bag for a duration of 3 h. To avoid contamination by odours from textiles or washing detergents, we will ask you to remove all your clothes but your underwear. You will get into the bag, it will be closed around your neck (this means, all your body but your head will be in the bag) and you will lay down on a bed and be covered with a blanket. We will then attach the pumps and columns to your bag, as well as a tube for introducing synthetic air (same as “normal” air, but without any odours) close to your shoulder. There will be no mosquitoes involved; it is only you in the bag! To keep you busy during the 3 h collection period, you will be able choose from a wide variety of movies or listen to a podcast. We will check on you regularly during the collection period.

Blood type

We are also interested in correlating the collected data with your ABO blood type, in case you know it. If you do, please bring a document stating your blood type (*e.g.* blood donation ID card).

Handling of Personal Data

To guarantee the protection of your personal data, your personal ID will be disentangled from the data set directly after obtaining all parts. A pseudonym will be used hereafter to analyse and compare different aspects of the data set across participants and for further publication of the study. The key that connects personal ID and pseudonym is stored separately and will be destroyed once the study is published.

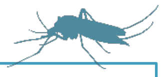

Other useful tips:

- Bring your headphones for watching a movie or listening to podcasts. The pumps can be a bit noisy.
- Do not drink too much before the odour collection since you cannot leave your bag for 3 h.
- Bring your normal shower equipment; we have showers in the building. And trust us, you want to have a shower after 3 h in a plastic bag ;)
